# Temporal and cross-site validation of an AI system for self-harm detection

**DOI:** 10.1371/journal.pdig.0001667

**Published:** 2026-09-11

**Authors:** Vlada Rozova, Liuliu Chen, Katrina Witt, Mike Conway, Jo Robinson, Karin Verspoor

**Affiliations:** 1 Department of Data Science and AI, Monash University, Melbourne, Australia; 2 School of Computing and Information Systems, University of Melbourne, Melbourne, Australia; 3 Centre for Digital Transformation of Health, University of Melbourne, Melbourne, Australia; 4 Centre for Youth Mental Health, The University of Melbourne, Melbourne, Australia; 5 Orygen, Melbourne, Australia; 6 School of Computing Technologies, RMIT University, Melbourne, Australia; North Carolina A&T State University: North Carolina Agricultural and Technical State University, UNITED STATES OF AMERICA

## Abstract

Adequate self-harm surveillance is a key part of suicide prevention. Our previous research demonstrated that an artificial intelligence (AI)-based system could effectively detect self-harm in emergency department triage notes. However, the system was developed using data from a single hospital, raising concerns about its generalisability. Here, we aim to validate the system prospectively and externally to better understand its portability across hospitals. We leveraged emergency department data from two Australian hospitals, with free-text triage notes manually annotated for self-harm. The AI system was developed using data from a major metropolitan hospital in Melbourne, spanning 2012–2017. The system combined extensive text normalisation with a self-harm classification model utilising 1931 selected features. We prospectively validated the model on 329,655 triage notes from the same hospital over the following four years. For external validation, we used 316,877 triage notes from 2012 to 2021 from a regional hospital 150 km outside Melbourne. On the test set, the model achieved an area under the precision-recall curve (PR AUC) of 0.84 with a 95% confidence interval of [0.82, 0.86]. This performance remained stable at the development site with a PR AUC of 0.84, 95% CI [0.83, 0.85]. When applied in the regional context, the model’s ability to distinguish self-harm cases declined, resulting in an overall PR AUC of 0.78, 95% CI [0.77, 0.79]. While the text normalisation component was equally effective across the datasets, our analysis revealed that in regional settings, self-harm presentations are more likely to involve medication ingestion. At the metropolitan hospital, the AI system for self-harm detection maintained its epidemiological utility. At the regional hospital, the text normalisation process was effective, but performance was unstable primarily due to linguistic domain shift and differences in self-harm presentations.

## Author summary

We tackle a practical challenge in clinical AI: ensuring text-based systems work well across different healthcare settings. Local documentation practices, semantics, local jargon, and patient demographics greatly affect how well an AI model works in new environments. We built an AI system for self-harm detection in emergency departments using real-world data from a large metropolitan hospital in Australia. We assessed the temporal stability and regional variability of the tool, comparing its performance at the development site and a regional hospital. The results showed the AI system is suitable for public health use at the metropolitan hospital. However, performance was unstable at the regional hospital due to linguistic variation and differences in methods patients use to self-harm.

## Introduction

Globally, over one million people die by suicide each year [1]. Suicide remains the leading cause of death for Australians aged 15–44, and in 2024 alone, suicide claimed 3,307 lives across all ages [2]. Previous self-harm is the strongest risk factor for future suicide [3]. Given the strong link between self-harm – particularly when frequently repeated – and suicide [4], establishing robust self-harm surveillance systems is crucial. These systems can act as an early warning mechanism for suicide and help evaluate national suicide prevention strategies [5,6].

In Australia, emergency departments (ED) often serve as the primary, and sometimes sole, point of contact with healthcare services for patients following an episode of non-fatal self-harm. Self-harm is defined as any act of self-injury and/or self-poisoning irrespective of the degree of suicidal intent and/or other motivations [7]. In contrast, suicidal ideation refers to having suicidal thoughts or intention to act on those thoughts without current actions [8]. It is more common than self-harm and is also closely linked to suicide. The accurate detection and monitoring of these events are crucial for public health initiatives, as they provide insight into self-harm prevalence, enable assessment of healthcare practices related to preventing repeated self-harm, and help estimate regional variation [9].

However, relying solely on diagnostic coding within ED records often results in inadequate capture of self-harm incidents [10]. A recent systematic review revealed an average sensitivity of 35.5% for intentional self-injuries and a positive predictive value of 63.8% [11]. Furthermore, in Canada and Australia, self-harm coding in emergency departments was reported to have a sensitivity below 40% [12,13]. Adding unknown intent and accidental poisoning codes improved sensitivity to 44.8% but still significantly underestimated the true incidence rates. To improve the ascertainment of self-harm cases, nursing triage notes present a valuable, yet underutilised, source of information. These brief texts typically detail the reason for the ED visit, patient symptoms, physical appearance, and relevant medical history, potentially offering insights into the reasons and methods of self-harm.

We have previously developed an artificial intelligence (AI) system that uses natural language processing (NLP) and machine learning (ML) to detect self-harm from ED triage notes [14]. Our purpose was twofold: a) to flag individual patient presentations that followed an episode of self-harm and b) to provide an estimate of the number of self-harm cases in the population. A near-perfect performance is essential for clinical application. For epidemiological use, the positive predictive value (PPV) and sensitivity are merely required to be balanced, since the overall trend is more important than accurately detecting individual cases.

The AI tool was developed using data from the Royal Melbourne Hospital (RMH), one of the largest publicly funded hospitals in metropolitan Melbourne, Victoria, Australia. The application was limited to ED presentations of patients aged nine and older. This was partly due to the difficulty in discerning the intent behind self-harm in younger children. Additionally, RMH sees very few paediatric cases due to its proximity to the Royal Children’s Hospital, which has its own emergency department.

Triage notes manually annotated by suicide prevention experts revealed less than 3% of cases involved self-harm or suicidal ideation [15]. Despite such a data imbalance, our AI system demonstrated strong performance in detecting self-harm cases, though some confusion with suicidal ideation was noted. The system was deployed at Orygen, a youth mental health organisation, in Australia to support a self-harm monitoring system across the state of Victoria [6]. This study aims to confirm whether the system can be used prospectively at the development site and applied to other hospitals across the state.

## Background and significance

Thorough validation of AI-based systems in healthcare is especially important given the potentially devastating consequences of errors. In hospital settings, real-world performance often degrades due to various factors, ranging from differing patient demographics to site-specific protocols and data entry or management systems [16,17]. Prospective and external validation studies are used extensively to ensure ML models generalise to unseen data [18–21]. Most validation studies are performed retrospectively to minimise the risks of premature deployment. The Epic Sepsis Model, a proprietary sepsis prediction model implemented by Epic in hundreds of US hospitals, has been an exception. The ease of integration within electronic health records allows researchers across multiple sites to validate the model locally and in real time [22]. Yet studies report limited discrimination of sepsis onset [23,24] and poor timeliness compared to traditional scoring algorithms [25].

Sociolinguistic variations across communities further contribute to dataset shift when analysing text-based documentation [26]. NLP models are additionally susceptible to performance issues due to variations in document structure and language [27], with extrinsic validation identified as critical [28]. Even with all other factors equal, different vocabulary and phrases used to describe conditions may severely affect the results. Approaches to validating NLP-based models include measuring the semantic similarity of corpora [29] and cross-checking models’ performance trained on different datasets [30]. At least some customisation is often required, either adjusting the text normalisation process [31,32] or re-training the ML classifier [33].

This study aims to evaluate the temporal robustness of the AI system at the development site and assess its cross-site portability. We explore how variations in local vocabulary and linguistic patterns influence differences in performance over time.

For prospective validation, we look at the next four years (2018–2021) of ED presentations at RMH. For external assessment, we obtained data from the Latrobe Regional Hospital (LRH), one of the eight hospitals participating in the state-wide self-harm monitoring system [6]. LRH is a public hospital located in regional Victoria, Australia, approximately 150 km from the nearest large city, Melbourne. Its catchment area is broadly representative in terms of age and gender but with a greater than average proportion of residents who identify as Aboriginal and/or Torres Strait Islander and a greater than average proportion of residents born in Australia.

As described in our previous work, nursing triage notes are often documented hastily and contain higher than typical levels of spelling mistakes. An example of a note could be, *“Taken 20x5mg diaz. O/A GSC 15, drowsy but tearfull. PHx dep[ression. Multiple superficial lacs on L) arm from 2/7 ago.”* Extensive use of ED-specific terminology, abbreviations, and slang is also characteristic of the notes. Normalising such text presents a challenging task. Our AI system consists of two stages: text normalisation and classification.

In the current work, we aim to track changes in performance and pinpoint potential reasons for disparities by answering the following questions:

- Was the population of patients similar (i.e., were observable variables like age, sex, and arrival mode equally distributed)?
- Was similar language used to record the information (i.e., is the text normalisation component equally effective)?
- Were similar expressions used to describe self-harm (i.e., does the classification model achieve comparable performance)?

Both components of our system can be customised to new data. However, text normalisation requires a human in the loop to review the results of spelling correction, and the classification model requires data to be expert-annotated for self-harm. As such, we explore whether tuning the first component without obtaining self-harm labels can improve the downstream task performance.

## Methods

### Data sources

In this study, we build on the 2012–2018 dataset compiled from ED presentations to RMH for the previous work [14], extending it to encompass the ten years 2012–2021. ED presentations to LRH spanned the same period (2012–2021). Records were directly extracted from the respective hospital information systems. Data fields included age, gender, time and mode of arrival, and a triage comment based on the initial nursing assessment. The extracts contained 728,995 rows of data from RMH and 361,997 rows of data from LRH.

The study received ethical approval from the Melbourne Health Human Research Ethics Committee (HREC; 2017.342).

### Data annotation

All data from RMH and LRH were annotated by a team of trained researchers and experts in suicide prevention at Orygen, Australia. The textual component of each ED presentation was labelled as self-harm, suicidal ideation, or neither. Only presentations linked to a current episode of self-harm or suicidal ideation were labelled positive. A note indicating a history of self-harm or suicidal ideation alone would be annotated as negative. The annotators used the same definitions across the two sites and were blinded to other fields during the annotation process.

We estimated that annotation and subsequent audits take up to 20 person-hours per 1,000 notes. Due to the large volume of data, the notes were annotated by multiple annotators over a period of several years. As reported in our previous study [14], the inter-annotator agreement for self-harm, calculated as Cohen’s kappa, was 0.91.

### Classification task

We developed a self-harm classification model to detect self-harm at triage in ED settings. We trained the model to detect ED triage notes annotated as positive for self-harm; all notes negative for self-harm, including those positive for suicidal ideation, formed the negative class. The developed model applies only to patients aged nine and older and only to the textual component of an ED presentation.

### Dataset preparation

Annotated data extracts were harmonised to have the same column names; sex and arrival mode were standardised to a set of predefined categories. The RMH dataset contained 36 duplicated rows, which were removed. We also removed ED presentations of patients aged eight years or younger; these amounted to 193 rows in the RMH dataset and 45,120 rows in the LRH dataset. This resulted in 728,766 rows of RMH data and 316,877 rows of LRH data.

**Fig 1:**
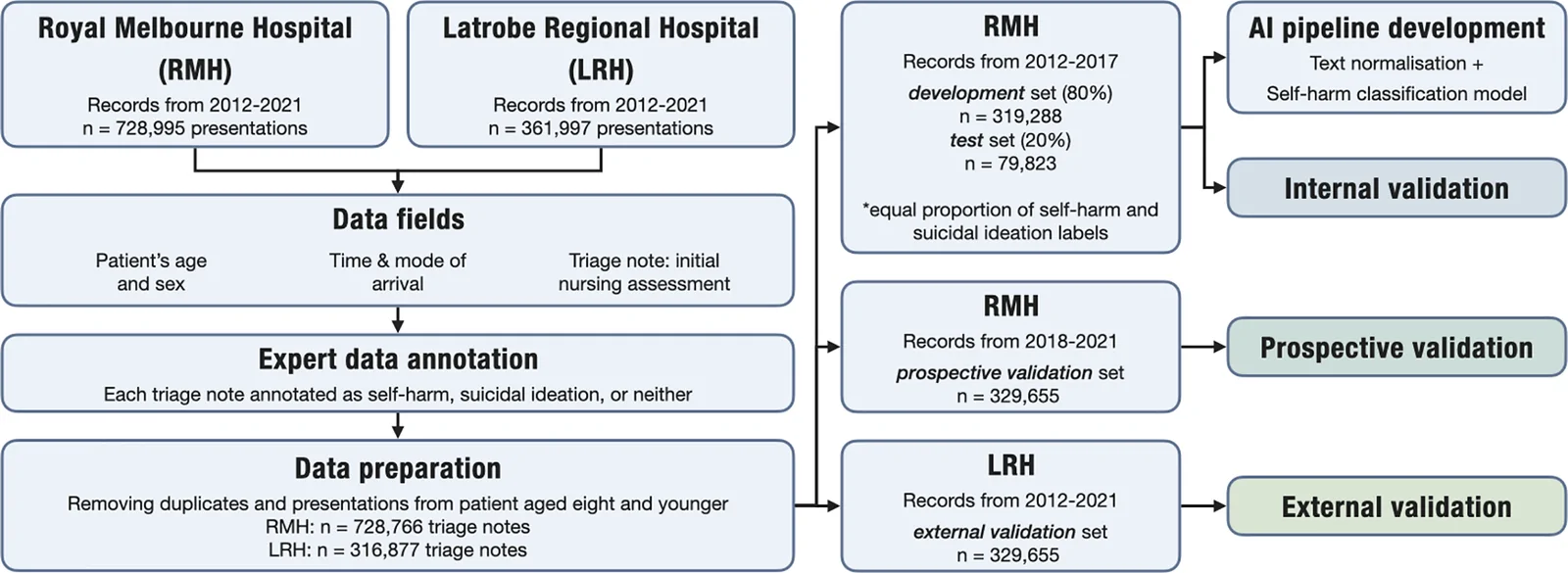
Datasets of ED presentations used in this study. Triage notes used for the development of the AI system for self-harm detection (80% of ED presentations at RMH in 2012-2017; *development* set), internal validation (20% of ED presentations at RMH in 2012-2017; *test* set), prospective validation (all ED presentations at RMH in 2018-2021), and external validation (all ED presentations at LRH in 2012-2021).

Of the RMH records from 2012-2017, we used 80% (n = 319,288) for model development and 20% (n = 79,823) for internal validation (testing), ensuring equal proportions of self-harm and suicidal ideation labels. We reserved RMH data from 2018-2021 for prospective validation (n = 329,655) and used all ten years of LRH data (n = 316,877) to assess model performance at the external site (Fig 1). Throughout the paper, we refer to data used for model development, internal, prospective, and external validation as *development*, *test*, *prospective validation*, and *external validation* datasets.

### Text normalisation

To support tokenisation and spelling correction, we first collated a domain-specific vocabulary of words and abbreviations relevant to the ED context. We combined multiple resources, then filtered out words that did not appear in the *development* set (S1 Text). The final vocabulary includes 20,115 words.

Using the learned domain-specific vocabulary, for each out-of-vocabulary word in the *development* set, we attempted to find its correct spelling (S2 Text). We built a dictionary of misspelled words and their correct spellings, yielding 43,909 pairs. Since finding candidates for a misspelled word is computationally expensive, we opted for a static approach and used the learned dictionary to correct spelling in unseen triage notes.

#### Custom pre-processing.

First, the text was converted to lowercase. Based on the *development* set, we developed a set of regular expressions to resolve commonly used shorthands, including *“++ve”* → *“positive”*, *“r/ship”* → *“relationship”*, *“L)”* → *“left”*.

#### Tokenisation.

We used the domain-specific vocabulary to introduce whitespaces into composite tokens, for example, *“warm/pink/dry”* → *“warm / pink / dry”*, *“20x5mg”* → *“20 x 5 mg”*, while preserving time, dates, and vitals, e.g., not adding spaces to *“2/7 ago”*.

#### Spelling correction.

We used the learned dictionary of misspellings to correct known misspellings, such as *“aggitated”* → *“agitated”*, *“dizzyness”* → *“dizziness”*, *“intermittant”* → *“intermittent”*.

#### Slang replacement.

We replaced commonly used alternative names for relevant drugs, such as *“fent”* → *“fentanyl”*, *“diaz”* → *“diazepam”*.

#### Filtering.

Lastly, we filtered out tokens that do not contain any letters, for example, removing *“36.7”* but retaining *“t2dm”*, a common notation for type 2 diabetes mellitus.

### Development of the classification model

We transformed text into its numerical representation using a term frequency-inverse document frequency (TF-IDF) vectoriser, which removed stop words and split tokens by whitespace. We retained features above a given percentile based on the chi-squared statistics. Other parameters of the vectoriser and the percentile value for feature selection were defined during hyperparameter tuning.

We compared four algorithms: multinomial Naive Bayes, Logistic Regression, Random Forest, and three implementations of the gradient boosting classifier (S3 Text). The three components were jointly optimised as a single model, including tuning hyperparameters of the vectoriser and the feature selector, to predict the probability of self-harm.

To select the best-performing model, we used a 5x3-fold nested cross-validation on the *development* set. The inner loop tuned hyperparameters optimising for negative log loss. The outer loop compared models based on negative log loss, the area under the receiver operator characteristic curve (ROC AUC), and the precision-recall curve (PR AUC) of the positive class as secondary metrics.

For final hyperparameter tuning, we used a 5-fold cross-validation on the *development* set, optimising for PR AUC. This choice was guided by a significant class imbalance, where PR AUC is known to better reflect the model’s classification ability on the minority class.

To calibrate the model, we used a 5x3-fold nested cross-validation on the *development* set. In the inner loop, the base estimator was fitted to the training data and calibrated on the testing data. The three fitted classifier and calibrator pairs were combined into an ensemble model, with the output being the average predicted probability of all pairs. We used the outer loop to compare uncalibrated model outputs with those calibrated using isotonic scaling. We examined the calibration curves and chose the version with the higher PR AUC.

Finally, for each fold, we calculated the optimal probability threshold by maximising the F1 score – the point on the PR curve that best balances precision and recall. The average value was used as the final probability threshold. The threshold was treated as a model hyperparameter and once tuned, remained unchanged during both prospective and external model validation.

The final classification model was retrained on the full *development* set.

### Performance evaluation

For model comparison and selection, we calculated negative log loss, ROC AUC, and PR AUC. We also plotted ROC and PR curves to supplement the analysis. To assess model calibration, we examined calibration curves plotting the fraction of positive samples against the mean predicted probability. Finally, we evaluated the model’s classification ability by examining the confusion matrix and calculating positive predicted value (PPV), sensitivity, and specificity.

Throughout model development, all metrics were calculated using the same 5-fold cross-validation strategy and are reported as the mean plus or minus standard deviation. For internal, prospective, and external validation, all estimates are reported as the mean with 95% confidence intervals derived from 1000 bootstrap iterations. To track performance changes over time, we grouped triage notes by quarter (starting from January) based on the date of arrival to ED.

### Linguistic differences

#### Vocabulary comparison.

Let us refer to the set of all valid tokens in the normalised notes from the *development* set as the *reference vocabulary*. We compared the language of triage notes against the *reference vocabulary* by calculating the proportion of shared tokens. We also report the total number of unique, valid tokens before and after text normalisation and the extent to which the normalisation process reduced dimensionality.

#### Divergence analysis of selected features.

We compared distributions of selected features per quarter across each evaluation dataset with the entire *development* set. We calculated pairwise Jensen-Shannon (JS) divergence using raw counts and TF-IDF representations to capture the difference in both raw lexicon usage and importance-weighted representation. JS divergence values closer to 0 indicate similarity, while values >0.2 indicate a substantial shift. We further analysed the per-feature contribution to explore if the divergence was concentrated on specific features.

#### SHAP feature analysis.

To analyse how lexical features influenced model predictions across datasets, we applied SHapley Additive Explanations (SHAP) to the calibrated ensemble model. For each base learner, SHAP values were calculated for the selected features and averaged across learners to derive global importance estimates.

We used SHAP waterfall plots to illustrate the model’s decision-making process. Each waterfall plot displays the contribution of individual features towards the final prediction (S4 Text).

For more information on implementation, please refer to the project GitHub repository: https://github.com/vlada-rozova/self-harm-triage-notes.

## Results

### Comparison of the two hospitals

As mentioned in the introduction, multiple factors contribute to performance variation. Table 1 highlights the key differences between the two hospitals. One can easily see that the catchment areas and the number of ED presentations vary greatly — RMH sees at least twice as many patients per year as LRH. RMH also has a mental health team providing 24/7 care in the ED. Although patient populations are similar in terms of age and sex distribution, more patients in regional areas rely on themselves or community support to reach the ED rather than road ambulance services.

**Table 1:** Comparison of the two healthcare providers. Geographic differences between the hospitals and patient population statistics from ED presentations between 2012 and 2021.

	RMH 2012–2021 (n = 728,766)	LRH 2012–2021 (n = 316,877)
Funding	Public	Public
Location	Metropolitan	Regional
Catchment area	137 sq.km	42,000 sq.km
Population served	550,000	300,000
Proportion of Indigenous patients	Approx. 1.6%	Approx. 1.9%
Emergency Mental Health team	Yes	No
Number of ED presentations per year		
All patients	72895 (± 9526)	35963 (± 4613)
Nine years and over	72877 (± 9517)	31688 (± 4326)
Age	48 (± 21)	47 (± 23)
Sex		
Female	48%	52%
Male	52%	48%
Intersex	<1%	<1%
Unknown	<1%	<1%
Missing values	n = 0	n = 0
Arrival mode		
Self / community	23%	47%
Road ambulance	35%	29%
Private ambulance	1%	2%
Air ambulance	<1%	<1%
Police	<1%	<1%
Undertaker	<1%	0%
Other	39%	22%
Missing values	n = 8	n = 89
Annotations		
Suicidal ideation	1.5%	1.9%
Self-harm	1.4%	1.7%
Triage note		
Character length	130 (± 56)	277 (± 126)
Empty note	n = 6072	n = 62

**Fig 2:**
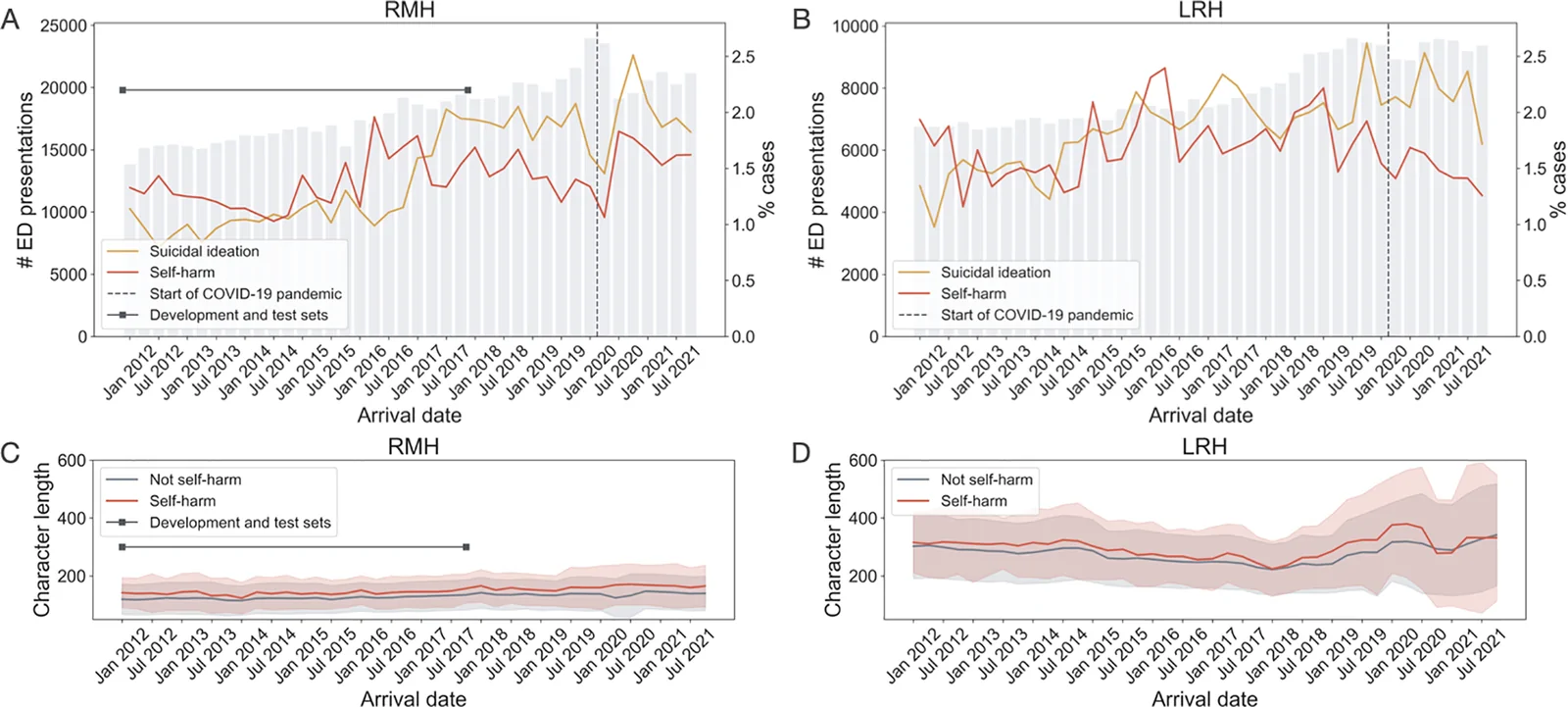
ED presentations and note length. **(a-b)** Number of ED presentations from 2012 to 2021 at RMH (left) and LRH (right). Grey bars show the total number of ED presentations. Prevalence of self-harm and suicidal ideation cases are shown in red and orange. The dashed vertical line indicates the start of the COVID-19 pandemic in Australia. **(c-d)** Character length of ED triage notes at RMH (left) and LRH (right). Triage notes annotated as positive for self-harm are shown in red, controls are shown in grey. Solid lines show the average length per quarter and the shaded areas indicate the range as measured by standard deviation. Triage notes were grouped into quarters based on arrival dates. The first six years of RMH data were included in the *development* and *test* sets.

The overall proportion of self-harm cases over the ten years at RMH vs. LRH was 1.4% and 1.7%, respectively. The proportion of suicidal ideation cases was only slightly higher, 1.5% vs. 1.9%. The number of ED presentations per quarter at each hospital and the trends in self-harm and suicidal ideation cases over time are shown in Fig 2a–b.

Triage notes varied in format depending on the information system, some being in uppercase. The typical length of triage notes indicates how much information is documented during ED encounters (Fig 2c–d). Compared to the *development* set, triage notes at RMH have remained the same length. At LRH, triage notes, on average, were at least twice as long (see Table 1). At LRH, the length shortened during the pandemic and at both sites the notes were less detailed during this period.

### AI system for self-harm detection

**Fig 3:**
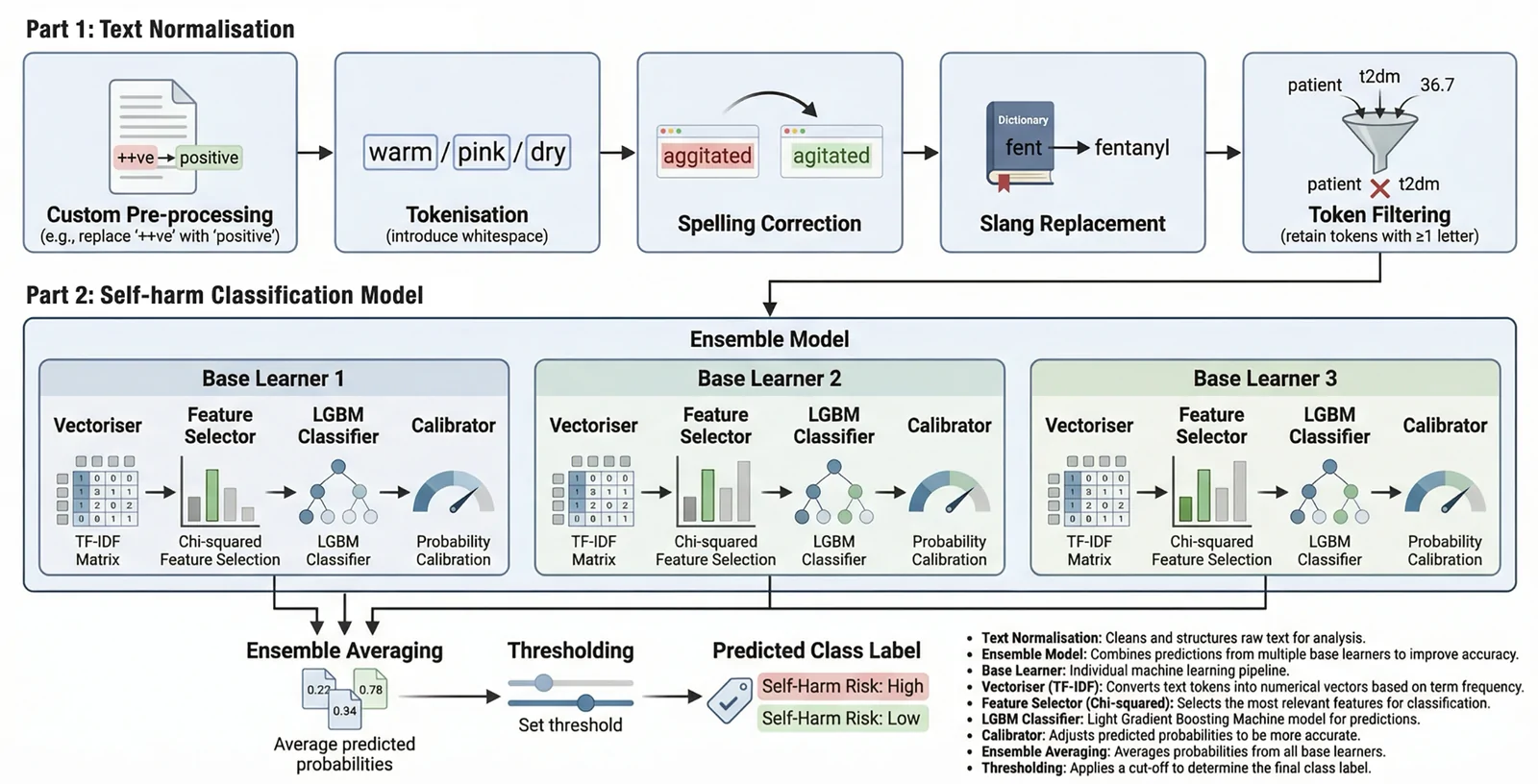
The AI system for self-harm detection. The system has two components: text normalisation and a self-harm classification model. Text normalisation consists of custom pre-processing, tokenisation, spelling correction, slang replacement, and token filtering. The classification model is an ensemble of three base learners, each including a vectoriser, a feature selector, and a LGBM classifier. The figure was created using the FigureLabs illustrator.

The full AI system for self-harm detection in triage notes is presented in Fig 3.

#### Text normalisation.

Passing a triage note through all steps of the text normalisation component resulted, for example, in the following transformation:

“*Taken 20x5mg diaz. O/A GSC 15, drowsy but tearfull. PHx dep[ression. Multiple superficial lacs on L) arm from 2/7 ago*” → “*taken x mg diazepam o/a gcs drowsy but tearful phx depression multiple superficial lacs on left arm from ago*”

#### Self-harm classification model.

Our self-harm classification model was developed on normalised triage notes from the *development* set. Comparing different classifiers, the Light Gradient Boosting Machine (LGBM) classifier achieved the best results across all metrics. The best configuration involved calculating TF-IDF values for unigrams, bigrams, and trigrams, retaining the top 2% of features, and passing them into the LGBM classifier (for the full list of hyperparameters, see S5 Text). The uncalibrated model achieved PR AUC of 0.850 (± 0.009) in cross-validation (S1 Table). Calibration further improved performance, increasing PR AUC to 0.853 (± 0.009) (S1 Table, S1 Fig). Across folds, the optimal probability thresholds were equal to 0.32 (± 0.06). The resulting PPV, sensitivity, and specificity are reproduced in Table 2.

**Table 2:** Overall performance of the AI system for self-harm detection. *Development* set scores are reported as mean (± standard deviation); *test*, *prospective validation*, and *external validation* results are reported as mean with 95% confidence intervals (95% CI).

	Development set (n = 319,288)	Test set (n = 79,823)	Prospective validation set (n = 329,655)	External validation set (n = 316,877)
ROC AUC	0.99 (± 0.0)	0.99, 95% CI [0.99, 0.99]	0.99, 95% CI [0.99, 0.99]	0.99, 95% CI [0.98, 0.99]
PR AUC	0.85 (± 0.01)	0.84, 95% CI [0.82, 0.86]	0.84, 95% CI [0.83, 0.85]	0.78, 95% CI [0.77, 0.79]
PPV	0.79 (± 0.01)	0.79, 95% CI [0.77, 0.81]	0.77, 95% CI [0.76, 0.78]	0.71, 95% CI [0.70, 0.72]
Sensitivity	0.79 (± 0.02)	0.79, 95% CI [0.77, 0.82]	0.76, 95% CI [0.75, 0.77]	0.73, 95% CI [0.72, 0.75]
Specificity	1.0 (± 0.0)	1.0, 95% CI [1.0, 1.0]	1.0, 95% CI [1.0, 1.0]	1.0, 95% CI [1.0, 1.0]

The final model was an ensemble of three base learners: each learner consisted of a vectoriser, a feature selector, an LGBM classifier, and a calibrator. These base learners selected sets of 1355, 1347, and 1315 features, totalling 1931 unique features. Predicted probabilities were averaged across the base learners, with the probability threshold of 0.32 applied to convert these probabilities into class labels (Fig 3).

#### Additional experiments.

We examined the possibility of selecting more features during feature extraction. Originally, we set the limit of 3% during hyperparameter search for feature selection, with 2% found to be the optimal value. Increasing the upper limit to 15% resulted in an 11% cut-off selecting a total of 10,706 features. Despite a five-fold increase in the number of features, the PR AUC of 0.86 (± 0.01) suggested only marginal improvement in model performance (S1 Table).

We also conducted an ablation study to evaluate the extent to which text normalisation improved downstream classification. We followed the same steps of the model development process as above, except this time using the raw triage notes from the *development* set. The best PR AUC was 0.81 (± 0.01), significantly lower than for normalised triage notes (S1 Table).

### Model evaluation

As outlined in the methods, the self-harm classification model was trained on the *development* set. We then evaluated it internally using the *test* set, which contained unseen data from the same hospital and time period. The model remained well-calibrated and achieved a PR AUC of 0.84 with a 95% confidence interval of [0.82, 0.86] (S2a-c Fig).

Table 2 reproduces these results along with PPV, sensitivity, and specificity. The *test* set performance is consistent with estimates from the *development* set. Equal PPV and sensitivity imply the probability cut-off produces a comparable number of false positives and negatives, as illustrated in a confusion matrix in S2e Fig. This is important for the model’s epidemiological application. A specificity close to 1.0 stems from a significant class imbalance, with less than 2% of positive cases.

We also investigated the confusion between self-harm and suicidal ideation. Out of the 227 false-positive notes, 53 were labelled as positive for suicidal ideation by annotators. This suggests approximately 23% of notes classified as self-harm may instead be positive for suicidal ideation (S2f Fig).

### Temporal and cross-site validation

The study estimates the temporal and geographic robustness of the AI system for self-harm detection through prospective and external validation. The *development* and *test* datasets comprised ED presentations at RMH between 2012 and 2017 inclusive. Prospective validation involved records from the same site over the four subsequent years, 2018–2021. The *external validation* set spanned ten years from 2012 to 2021 at LRH.

#### Prospective validation at RMH in 2018–2021.

In the original study, prospective validation was limited to 2018 [14]. When running the system on data from 2018 to 2021, all metrics except sensitivity remained comparable to the *test* set (Table 2, S3 Fig). Sensitivity slightly decreased from 0.79, 95% CI [0.77, 0.82] to 0.76, 95% CI [0.75, 0.77]. However, these overall estimates may not accurately reflect the temporal changes in performance.

**Fig 4:**
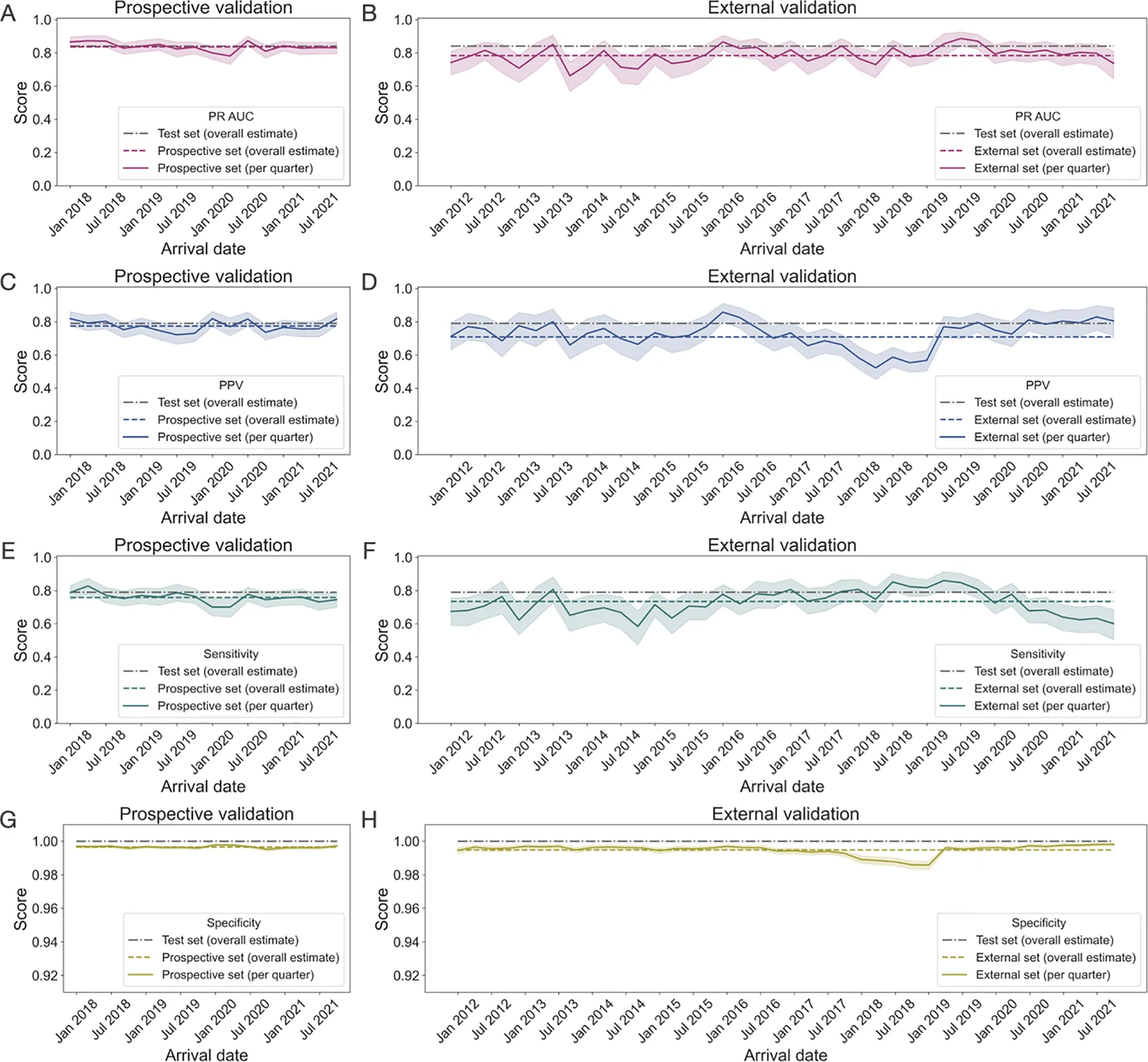
Performance of the AI system for self-harm detection over time. PR AUC **(a-b)**, PPV **(c-d)**, sensitivity **(e-f)**, and specificity (**g-h**; note the altered y-axis scale) on the *prospective validation* (left) and *external validation* (right) datasets. Dashed lines indicate estimates calculated over the full set of triage notes as reported in Table 2. Solid lines show mean scores with 95% confidence bands over time, where triage notes were grouped into quarters based on arrival dates.

We thus inspected changes in performance over time by grouping triage notes into quarters based on arrival dates. As outlined in the methods section, we bootstrapped each subset of three months and calculated PR AUC, PPV, sensitivity, and specificity. Fig 4 displays average scores per quarter along with 95% confidence bands and compares them to overall estimates from the *test* and *prospective validation* sets. PPV receded slightly in mid-2019 and sensitivity contracted around the first half of 2020, which coincided with the outbreak of the COVID-19 pandemic in Australia, but then stabilised. The AI system appears to maintain its epidemiological utility at the site of development.

#### External validation at LRH in 2012–2021.

Externally, performance noticeably declined with a PR AUC of 0.78, 95% CI [0.77, 0.79], and consequently lower PPV (0.71, 95% CI [0.70, 0.72]) and sensitivity (0.73, 95% CI [0.72, 0.74]) (Table 2, S4 Fig). Interestingly, predicted probabilities remain well-calibrated, and, at first glance, the selected probability threshold achieved a balanced PPV and sensitivity.

However, a closer examination of model performance quarter by quarter revealed significant deviations from the *test* set estimates. PPV dropped from early 2017 until early 2019 before recovering. Sensitivity largely fell below the *test* set estimate until mid-2015 and then declined again in the second half of 2020, continuing to decrease thereafter.

We investigated whether incorporating LRH data during training could remedy the diminished performance. We applied the original text normalisation to LRH triage notes from 2012 to 2017.

First, we retrained the model solely on LRH data, starting with presentations in 2012 and adding one year of data at a time. Only after incorporating all data from 2012 to 2017 did the model score a PR AUC of 0.85 (± 0.02). These experiments highlight the need for a substantial amount of local data to match performance at the development site, estimating 6,320 person-hours to gather six years’ worth of data at LRH.

Next, we incrementally added one year’s worth of LRH data to the *development* set and retrained the model. The best cross-validation PR AUC of 0.81 (± 0.01) was achieved when all six years of LRH data were combined with the original *development* set (S2 Table). Falling short of the original performance, these results indicate critical differences in underlying data. In the following sections, we examine whether these differences are inherent to the corpora or are an unintended effect of text normalisation.

### The effects of text normalisation

The triage note normalisation approach was developed using data from RMH. To assess its portability, we evaluated its impact on triage notes from both hospitals and over time. We compared the language of the *test*, *prospective*, and *external validation* sets to the *reference vocabulary*. Before text normalisation, the number of unique tokens was the highest in the *prospective validation* set (S5a–c Fig). In all three datasets, fewer than 50% of tokens were shared with the *reference vocabulary* (S5d–f Fig). After text normalisation, the number of unique tokens dropped to approximately 10,000 in all three datasets. In *prospective* and *external validation* datasets, the proportion of tokens shared with the *reference vocabulary* increased to around 75%. A sudden increase in language similarity seen in the *external validation* data around April 2019 coincides with the roll-out of a new information management system at LRH (S5f Fig). Text normalisation reduced the dimensionality of triage notes by about 50% in the *prospective validation* data, being slightly less effective on the *test* and *external validation* sets (S5g–i Fig). Taken together, these results do not reveal any dramatic differences in the underlying vocabulary that could explain the observed drop in performance.

### Corpus comparison analysis

To verify the portability of the AI system, we examined the distribution of tokens used as classifier inputs after text normalisation had been applied. As described above, the classification model was an ensemble of three base learners. Each base learner selected a set of features, adding up to 1931 unique features across the three sets. To assess their impact on model generalisation, we conducted two complementary analyses: (1) JS divergence analysis to quantify lexical distribution shift across datasets; (2) SHAP feature importance analysis to examine how model reliance on predictive features differs across corpora.

**Fig 5:**
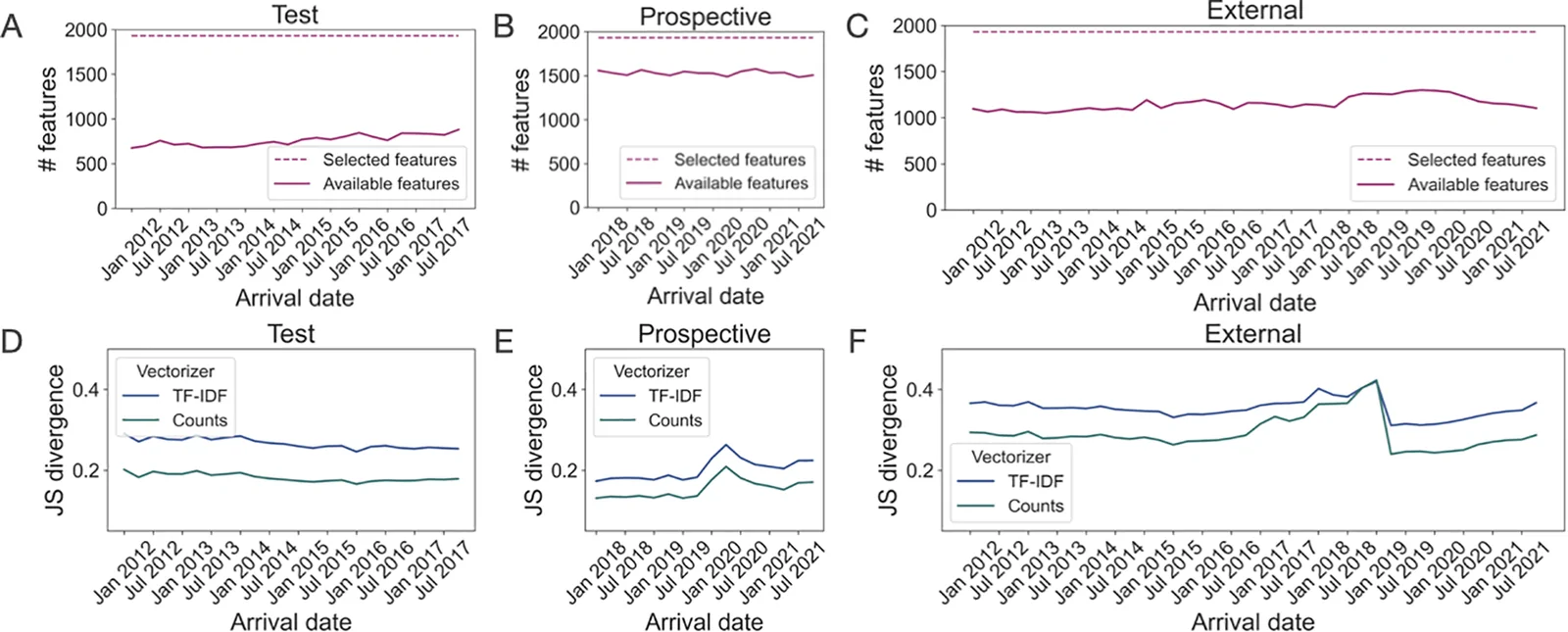
Distribution of selected features. **(a-c)** Features available after text normalisation in the *test* (left), *prospective validation* (centre), and *external validation* (right) datasets. Solid line shows the number of available features; dashed line indicates the combined 1931 features selected by the three base learners. **(d-f)** Jensen-Shannon divergence from the *development* set feature distribution, calculated as raw counts and TF-IDF scores. Changes in all metrics are shown over time, where triage notes were grouped into quarters based on arrival dates.

Fig 5 illustrates that while a sufficient number of selected tokens persisted in the notes, their distribution varied. In the *prospective validation* set, the divergence increased sharply in early 2020. The *external validation* set shows considerable divergence from the *development* set throughout the entire 10-year period. Consistently, the TF-IDF representation shows higher JS divergence values compared to the count representation. This suggests differences are more pronounced in relative term importance than raw frequency alone.

Table 3 summarises the frequency of the top 10 most divergent features across quarters using TF-IDF representations. The *external validation* set revealed a stronger contribution from medication-related and substance-use terms like *“panadol”* (an Australian brand name for paracetamol), *“etoh”* (clinical abbreviation for alcohol), and *“mg”* (unit).

**Table 3:** Top 10 features contributing to JS divergence. Features that most frequently occur across quarters in the top 20 features with the highest JS divergence value. Meaning: *“section 351”* (Victorian Mental Health Act 2014), *“o/a”* (on arrival), *“gcs”* (Glasgow Coma Scale), *“panadeine forte”* (an Australian brand name for paracetamol and codeine), *“ecatt”* (Enhanced Crisis Assessment and Treatment Team), *“si”* (suicidal ideation), *“panadol”* (an Australian brand name for paracetamol), *“etoh”* (clinical abbreviation for alcohol), *“loc”* (loss of consciousness), *“mg”* (unit).

Development vs. test set		Development vs. prospective validation set		Development vs. external validation set	
Feature	#quarters	Feature	#quarters	Feature	#quarters
section	22	o/a gcs	16	panadol	40
section351	17	ecatt	16	direct	40
o/a gcs	13	direct	16	etoh	40
panadeine forte	6	panadeine	16	o/a gcs	40
direct	6	taken	15	loc	40
ecatt	5	forte	14	taken	38
nil	5	chest pain	12	mg	33
violence	5	si	12	states	33
drug	4	chest	10	mum	31
forte	4	panadeine forte	10	approx hours	30

**Fig 6:**
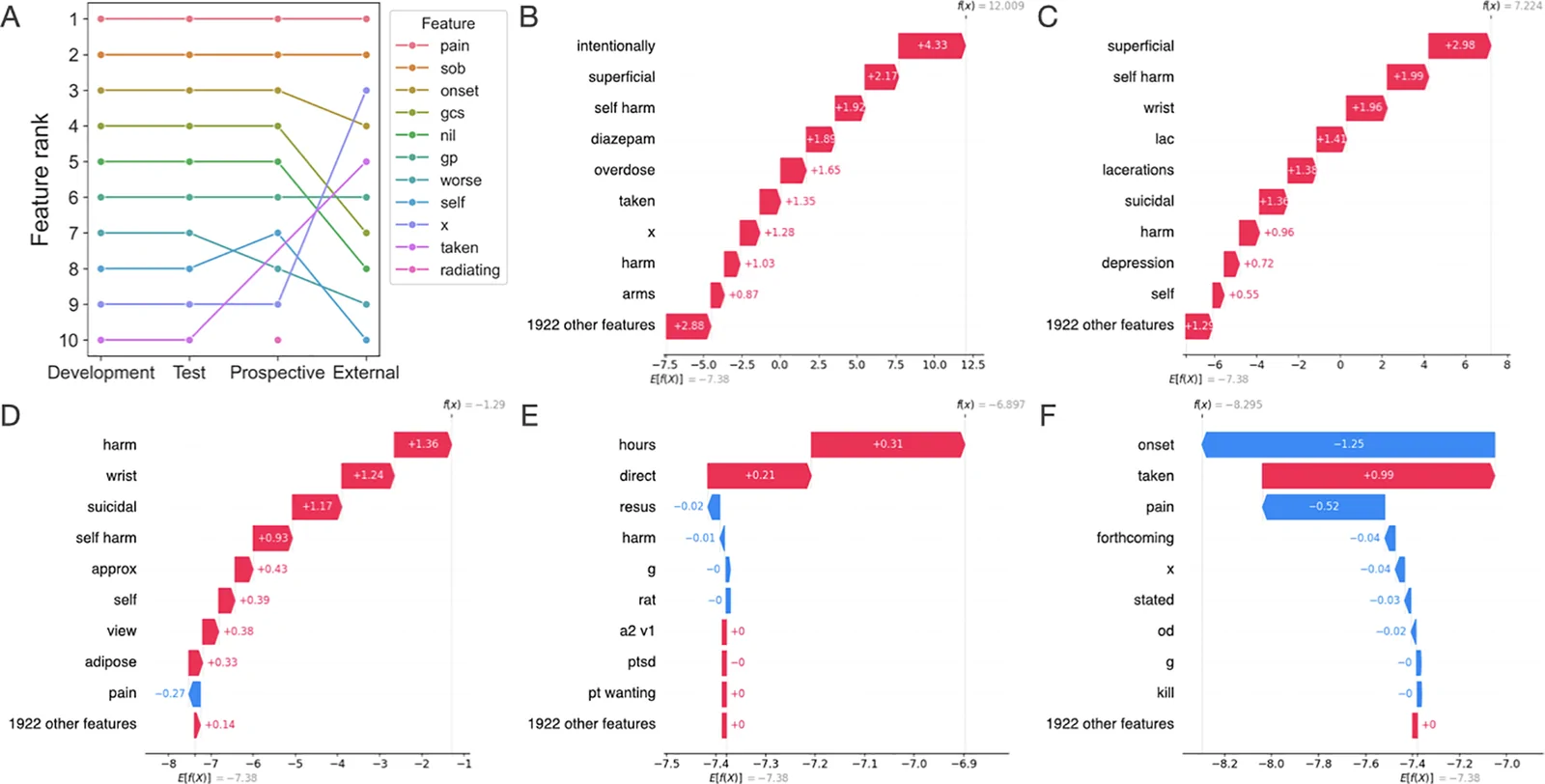
SHAP analysis results. **(a)** Top 10 features with the average highest global importance score and the persistence of the ranking across datasets. **(b-f)** SHAP waterfall plots for five examples with feature contributions towards final predictions. Blue bars show features weighing towards Not self-harm, red bars show features pushing the model towards Self-harm. Meaning: *“sob”* (shortness of breath), *“gcs”* (Glasgow Coma Scale), *“gp”* (general practitioner), *“x”* (multipler), *“lac”* (laceration), *“g”* (unit), *“ptsd”* (post-traumatic stress disorder), *“pt”* (patient), *“od”* (overdose).

The top 10 features with the highest average global importance within each dataset are shown in Fig 6a. These features predominantly consist of clinical terms like *“pain”*, *“sob”* (abbreviation for shortness of breath), *“onset”*, and *“gcs”* (Glasgow Coma Scale). Within each dataset, the RMH data exhibited remarkably similar importance patterns, indicating internal consistency. The *external validation* set shared many of these features, but their relative rankings varied noticeably.

Finally, we calculated the difference in feature importance between the *development* set and each evaluation dataset and ranked features by the magnitude of this difference (S3 Table). The greatest variation was observed between the *development* and *external validation* sets, with higher absolute differences for terms related to medication intake, e.g., *“x”* (multiplier), *“taken”*, *“mg”*, *“took”*. These shifts in feature importance align with the observed lexical divergence in the JS analysis.

Taken together, these observations reveal that in regional settings, both general complaints and self-harm presentations are more likely to involve medication ingestion. Furthermore, the results indicate self-poisoning, rather than self-injury, may be the more common primary mechanism of self-harm.

### Error analysis

Five anonymised examples are presented below, along with SHAP waterfall plots to illustrate the model’s decision-making process.

#### Example of a true positive.

Normalised triage note: *“pt taken oral overdose of x paracetamol and x diazepam pt reports he was intentionally trying to harm himself was in ed ago for same no vomiting no diarrhoea has some superficial self harm scratches on a arms”*

True label: Self-harm; predicted probability: 1.0; predicted label: Self-harm. This true positive shows the model performing as intended. Multiple explicit self-harm indicators (e.g., *“intentionally”*, *“self harm”*, *“overdose”*) are correctly identified as the strongest positive contributors, pushing the prediction well above the threshold (f(x) = 12.009). The originally misspelled *“intentially”* was successfully corrected during text normalisation (Fig 6b).

#### Example of a false positive.

Normalised triage note: “d/c from *** hospital yest post depressed suicidal thoughts and superficial lac ‘s to left wrist this am seen with knife by housemate no new lacerations flat affect hx bpd depression anxiety ptsd self harm”

True label: Not self-harm; predicted probability: 0.99; predicted label: Self-harm.

The note includes a rich psychiatric background: history of *“self harm”*, *“superficial lac”* (laceration), *“depression”*, etc., which are all associated strongly with SHAP contribution. However, the phrase *“no new lacs”* indicates no active self-harm at this presentation, which was considered a negative label during annotation. This case shows the model lacks the ability to negate and temporally reason, resulting in a false positive prediction (Fig 6c).

#### Example of a false negative.

Normalised triage note: *“biba hours self harm approx cm laceration left inner wrist adipose tissue on view per av minimal blood loss dressed with av so not viewed left thumb altered sensation phx suicidal ideation increased social stressors vitals stable pain”*

True label: Self-harm; predicted probability: 0.3215; predicted label: Not self-harm.

This false negative demonstrates an example close to the decision boundary. The predicted probability of 0.3215 falls short of the selected threshold of 0.3225. Despite clear signs of self-harm indicated in the note (e.g., *“self harm”* + 0.93), the strongly negative base value (E[f(x)] = -7.38) is only partially offset, resulting in a final score just under the threshold (Fig 6d).

#### Example of a false negative due to misspellings.

Normalised triage note: *“polupharmacy at hours agtiated on arrival heart rate sr on monitor direct to resus”*

True label: Self-harm; predicted probability: 0.0012; predicted label: Not self-harm.

This false negative example shows how misspellings, *“polupharmacy”* (polypharmacy) and *“agtiated”* (agitated), can lead to misclassification. The model failed to capture the two characteristic features of self-harm, showing no positive contribution on the SHAP waterfall plot. Instead, the model relies on weak contextual features (*“hours”* + 0.31, *“direct”* + 0.21) that are insufficient to overcome the strongly negative base value (E[f(x)] = -7.38), resulting in a predicted probability of 0.0012 (Fig 6e).

#### Example of a false negative due to non-specific terminology.

Normalised triage note: “onset dizziness stated taken aloe pain killers and has not eaten today recent d/c *** hosp not forthcoming with info phx autism”

True label: Self-harm; predicted probability: 0.0002; predicted label: Not self-harm.

This false negative shows how lay terminology, *“taken a lot of pain killers”* used here to describe intentional overdose, causes model failure. Moreover, *“onset”* (-1.25) significantly contributes to the negative prediction, as it is a common term in general ED triage notes unrelated to self-harm (Fig 6f).

Overall, these examples highlight that the model captures important self-harm-related features but is sensitive to lexical variation and standardisation of triage notes. Text normalisation and manual review of predictions close to the probability threshold could help improve prediction performance.

## Discussion

This study examines the temporal and cross-site portability of an NLP-based AI system for self-harm detection. We demonstrate that while the system retains its epidemiological utility, its accuracy decreases at the regional site. As discussed earlier, various environmental, technological, and organisational factors may continuously impact model performance. For example, while the presence of a 24/7 emergency mental health team at the RMH ED does not directly affect the quality of the notes—these are taken by nurses at the first encounter—there is an opportunity for training ED staff to better recognise and describe suicidal behaviours.

Our analysis of linguistic differences between hospitals reveals variations in self-harm presentations between metropolitan and regional populations. Features related to medication intake and substance consumption consistently rank higher in the regional hospital, suggesting self-poisoning as the more common method of self-harm.

A plethora of frameworks exist guiding the development and implementation of AI models in healthcare. The majority advise validating the candidate model in a new healthcare setting or on data from a different period [34]. This is usually sufficient to see that a model fails to generalise, as has been observed with the Epic Sepsis Model. However, a positive outcome of an external validation study does not guarantee the model’s universal generalisability [35].

Given the temporal nature of the factors affecting the data distribution, assessing the performance over time is especially important and can yield insights into the model’s underlying strengths and weaknesses. Recurring validation locally may be preferred over a one-off external validation [36]. Moreover, focus should be put on evaluating aspects relevant to the desired application rather than attempting to prove all-round generalisability [16]. A model yielding similar PPV and sensitivity may be valuable in the epidemiological context, even if its overall discriminatory ability is not appropriate for clinical use.

This study also highlights the importance of assessing regional variation. Most healthcare AI models are developed using data from major tertiary hospitals or research centres. Their portability to regional settings is limited not only by infrastructural and information systems aspects. Service availability, varying health literacy levels, and preference for community-based support can impact how patients interact with healthcare providers. In regional areas, patients may present with different risk factors or face unique socioeconomic challenges that influence their healthcare needs and outcomes.

Furthermore, the patterns of suicide-related behaviours might differ between urban and rural populations, affecting model performance. To deliver equitable care, AI models must be adaptable to these diverse contexts. Health systems must consider ongoing monitoring using application-specific metrics, recalibration, and tuning on local data before deploying NLP-based surveillance tools.

However, data annotation presents its own challenges. Appropriately trained personnel may be scarce in regional and remote areas. Conditions like self-harm and suicidal ideation are relatively subjective and often difficult to discern from the limited amount of information captured in nursing triage notes. Finally, in terms of human labour costs, collecting and annotating sufficient local data requires a significant time commitment.

Given these factors, we stress the importance of validating individual system components, comparing corpora to development data before annotation, and the shift towards temporal evaluation of AI models.

## Conclusion

The AI tool has been deployed to support a state-wide self-harm monitoring system, providing an opportunity for ongoing validation and refinement. We demonstrate that the AI system for self-harm detection maintains its utility at the original site when validated prospectively. Balanced PPV and sensitivity suggest the model could be used to reliably track the proportion of self-harm cases in the population as an operational epidemiological tool embedded in public health workflows.

As indicated by the error analysis, when searching for self-harm presentations, end-users should pay attention to the history of self-harm and consider potential confusion with suicidal ideation. Users may also choose to review cases with predicted probabilities just below the threshold. While manual review is still required, the algorithm dramatically reduces the amount of work, with less than 3% of notes assigned a probability above 0.1.

To enable its clinical use, a stronger ability to discern self-harm cases is required, particularly given the occasional confusion with suicidal ideation. Furthermore, analysis of the current workflow, the design of the optimal prediction delivery method, and considerations for the management of erroneous results are essential for clinical implementation of the system. A silent trial followed by a single-centre randomised controlled trial would be the initial steps in exploring the various factors involved in bringing an AI-based system into clinical practice.

Future work will focus on investigating the variations in self-harm presentations and exploring the use of more sophisticated AI methods to normalise data and extract discrete information from triage notes.

## Supporting information

S1 Fig Diagnostic plots for the *development* set using cross-validation. (a) Calibration curves of the uncalibrated and calibrated models. ROC curves (b) and PR curves (c) are shown as mean with standard deviation bands across five cross-validation folds.
(TIFF)

S2 Fig Diagnostic plots for the *test* set. (a-c) Calibration, ROC, and PR curves are shown as mean with 95% confidence bands derived from 1000 bootstrap iterations. (d) Distribution of predicted probabilities on the full dataset. Dashed vertical line indicates the selected probability threshold. (e-f) Confusion matrices showing the number of misclassified cases and confusion with suicidal ideation.
(TIFF)

S3 Fig Diagnostic plots for the *prospective validation* set. (a-c) Calibration, ROC, and PR curves are shown as mean with 95% confidence bands derived from 1000 bootstrap iterations. (d) Distribution of predicted probabilities on the full dataset. Dashed vertical line indicates the selected probability threshold. (e-f) Confusion matrices showing the number of misclassified cases and confusion with suicidal ideation.
(TIFF)

S4 Fig Diagnostic plots for the *external validation* set. (a-c) Calibration, ROC, and PR curves are shown as mean with 95% confidence bands derived from 1000 bootstrap iterations. (d) Distribution of predicted probabilities on the full dataset. Dashed vertical line indicates the selected probability threshold. (e-f) Confusion matrices showing the number of misclassified cases and confusion with suicidal ideation.
(TIFF)

S5 Fig The effect of text normalisation on triage notes from the *test* (left), *prospective validation* (centre), and *external validation* (right) datasets. (a-c) The number of unique tokens in each dataset before and after text normalisation was applied. (d-f) Proportion of tokens overlapping with the *reference vocabulary* before and after text normalisation. (g-i) The extent to which text normalisation reduced the dimensionality of data. Changes in all metrics are shown over time where triage notes were grouped into quarters based on arrival dates.
(TIFF)

S1 Table Model selection. Performance comparison on raw and normalised triage notes, with maximum top 3% and 15% of selected features, with and without probability calibration. PR AUC is reported as mean (± standard deviation) across five cross-validation folds.
(XLSX)

S2 Table Fine-tuning with LRH data. Results of model development and evaluation where normalised LRH triage notes were gradually incorporated into model training either with or without the original *development* set.
(XLSX)

S3 Table Top 10 features with the largest difference in importance scores. Features ranked by the magnitude of this difference compared to the *development* set.
(XLSX)

S1 Text Learning a domain-specific vocabulary. (DOCX)

S2 Text Learning a dictionary of misspellings. (DOCX)

S3 Text Model specifications. (DOCX)

S4 Text Error analysis. (DOCX)

S5 Text Model hyperparameters. (DOCX)
