# Supplementary material for: Temporal and cross-site validation of an AI system for self-harm detection
Source: PLOS Digit Health. 2026 Sep 11;5(9):e0001667. doi: 10.1371/journal.pdig.0001667 (PMC13567808; doi:10.1371/journal.pdig.0001667)
Supplement: Supplementary file 1 [file pdig.0001667.s014.pdf]

Dear reviewers,

On behalf of the team, I would like to thank you for the fantastic comments you have provided. We appreciate the time you took to review our manuscript and your thoughtful and thorough suggestions. We believe they substantially improved the quality of the work but we would be happy to address any remaining concerns.

Please find our responses to all your comments on the next page.

Best wishes,

Vlada Rozova

Reviewer #1:

1 The depth and necessity of the research background

Although the importance of self-injury monitoring in suicide prevention is mentioned in this paper, it does not fully link the shortcomings of traditional diagnostic coding with the advantages of NLP model. It is suggested to supplement the specific limitations of traditional methods (such as ICD coding) in self-injury detection (such as the rate of missing report and delay), and quote the latest research data (such as the empirical evidence of missing report of self-injury by diagnostic coding in recent years) to strengthen the necessity of this study.

Response

We have provided recent evidence demonstrating the limitations of using diagnostic coding to detect self-harm.

In Introduction:

"A recent systematic review revealed an average sensitivity of 35.5% for intentional self-injuries and a positive predictive value of 63.8% [10]. Furthermore, in Canada and Australia, self-harm coding in emergency departments was reported to have a sensitivity below 40% [11, 12]. Adding unknown intent and accidental poisoning codes improved sensitivity to 44.8% but still significantly underestimated the true incidence rates."

2 Reproducibility of model development details

The method part mentions that the model uses 644 selected features, but the specific method of feature selection (such as feature importance, chi-square test or L1 regularization) is not clear. The criteria of feature selection (such as threshold setting and exclusion criteria) and verification process (such as how to determine the optimal number of features in cross-verification) need to be supplemented to ensure the reproducibility of the method.

Response

Thank you for this comment. We have improved the design of our experiments to ensure more robust feature selection.

- Feature selection was based on chi-squared statistics.
- Vectorisation and feature selection are now jointly optimised together with the classifier to ensure optimal hyperparameter values (such as the threshold for selection).
- We ran an additional experiment to make sure adding more features would not improve model performance.

In Methods, Development of the classification model:

"We retained features above a given percentile based on the chi-squared statistics. Other parameters of the vectoriser and the percentile value for feature selection were defined during hyperparameter tuning."

In Results, AI system for self-harm detection, Self-harm classification model:

"The best configuration involved calculating TF-IDF values for unigrams, bigrams, and trigrams, retaining the top 2% of features, and passing them into the LGBM classifier (for the full list of hyperparameters, see Supplementary Materials, Model specifications)."

In Results, AI system for self-harm detection, Additional experiments:

"We examined the possibility of selecting more features during feature extraction. Originally, we set the limit of 3% during hyperparameter search for feature selection, with 2% found to be the optimal value. Increasing the upper limit to 15% resulted in an 11% cut-off selecting a total of 10,706 features. Despite a five-fold increase in the number of features, the PR AUC of 0.86 (± 0.01) suggested only marginal improvement in model performance (Table S1)."

3 transparency and basis of data preprocessing

The reason for excluding patients under 8 years old is "it is difficult to determine the intention of self-injury", but the specific proportion of self-injury events in this age group (such as the proportion of patients under 8 years old in the original data and the number of self-injury cases) or related research support is not provided. It is suggested to supplement the literature basis of exclusion criteria (such as clinical guidelines for the evaluation of children's self-injury intention) and discuss the potential impact of this exclusion on the generalization of the model (such as whether to limit the application of the model in pediatric scenes).

Response

Thank you for highlighting that it was not clear the model was only applicable to patients over eight years old.

- We have clarified that the application of the model was restricted to patients aged nine and older.
- We have re-iterated that the developed system was intended only for patients aged nine and older.
- We have also specified that in the RMH dataset, out of 700,000+ rows, there were only 193 records of patients aged eight and younger.

In Introduction:

"The AI tool was developed using data from the Royal Melbourne Hospital (RMH), one of the largest publicly funded hospitals in metropolitan Melbourne, Victoria, Australia. The application was limited to ED presentations of patients aged nine and older. This was partly due to the difficulty in discerning the intent behind self-harm in younger children. Additionally, RMH sees very few paediatric cases due to its proximity to the Royal Children's Hospital, which has its own emergency department."

In Methods, Classification task:

"The developed model applies only to patients aged nine and older and only to the textual component of an ED presentation."

In Methods, Data preparation:

"We also removed ED presentations of patients aged eight years or younger; these amounted to 193 rows in the RMH dataset and 45,120 rows in the LRH dataset. This resulted in 728,766 rows of RMH data and 316,877 rows of LRH data."

4. Selection basis of external verification hospitals

Regional hospital (LRH) was selected for external verification, but the key differences between LRH and the original hospital (RMH) in medical practice and recording habits (such as whether there is an emergency psychiatric team and the standardization of triage records) were not explained in detail. Table 2 compares some demographic information, but it is necessary to further discuss how these

differences (such as whether the non-psychiatric team of LRH leads to a more vague description of self-injury in triage records) may affect the model performance.

Response

We have clarified that Latrobe Regional Hospital was one of the sites participating in a broader research program.

We have further commented on the differences in note quality:

- We have clarified that triage notes used in this analysis are based on the initial nursing assessment.
- We have also commented on the potential effect of the presence of the mental health team in ED.

In Background & Significance:

"For external assessment, we obtained data from the Latrobe Regional Hospital (LRH), one of the eight hospitals participating in the state-wide self-harm monitoring system [5]."

In Methods, Data sources:

"Data fields included age, gender, time and mode of arrival, and a triage comment based on the initial nursing assessment."

In Results, Comparison of the two hospitals:

"Triage notes varied in format depending on the information system, some being in uppercase. The typical length of triage notes indicates how much information is documented during ED encounters (Figures 2c-d). Compared to the ***development*** *set, triage notes at RMH have remained the same length. At LRH, triage notes, on average, were at least twice as long (see Table 1). At LRH, the length shortened during the pandemic and at both sites the notes were less detailed during that period."*

In Discussion:

"For example, while the presence of a 24/7 emergency mental health team at the RMH ED does not directly affect the quality of the notes—these are taken by nurses at the first encounter—there is an opportunity for training ED staff to better recognise and describe suicidal behaviours."

5 Selection and interpretation of performance indicators

The study selected PR AUC, PPV, sensitivity and other indicators, but did not clearly explain why these indicators were selected (such as the reason why PR AUC is better than ROC AUC when the data is unbalanced). It is suggested to supplement the theoretical basis for the selection of indicators (for example, "the proportion of self-injury cases is less than 2%, and PR AUC can better reflect the performance of the model in a few categories"), and explain the practical significance of the specificity close to 1 (for example, the misjudgment rate of non-self-injury cases is extremely low).

Response

Thank you for pointing out the need for interpretation of selected metrics. We have clarified which metrics were used, when PR AUC was preferred and why. We have reported ROC AUC throughout the paper for completeness.

In Methods, Development of the classification model:

"To select the best-performing model, we used a 5x3-fold nested cross-validation on the ***development*** *set. The inner loop tuned hyperparameters optimising for negative log loss. The outer loop compared models based on negative log loss, the area under the receiver operator characteristic*

curve (ROC AUC), and the precision-recall curve (PR AUC) of the positive class as secondary metrics."

"For final hyperparameter tuning, we used a 5-fold cross-validation on the ***development*** *set, optimising for PR AUC. This choice was guided by a significant class imbalance, where PR AUC is known to better reflect the model's classification ability on the minority class."*

In Methods, Performance evaluation:

"For model comparison and selection, we calculated negative log loss, ROC AUC, and PR AUC. We also plotted ROC and PR curves to supplement the analysis."

6 mechanism analysis of time trend

Figure 2 shows that the performance of LRH decreased (sensitivity decreased to 0.68) after Q2, 2020, which was attributed to COVID-19 pandemic, but no specific evidence was provided (for example, the contents of triage records changed during the pandemic: was the record shorter and the terminology less standardized due to emergency? Has the type of self-injury changed? ）。 It is suggested to combine the adjustment of medical practice during the pandemic (such as remote triage and recording priority changes) and analyze its influence on text characteristics and model input.

Response

Our revised results no longer demonstrate a strong correlation with the start of COVID-19. We have added potential explanations where possible but given the current data we are unable to confirm them.

In Results, Temporal and cross-site validation, Prospective validation at RMH in 2018-2021:

"PPV receded slightly in mid-2019 and sensitivity contracted around the first half of 2020, which coincided with the outbreak of the COVID-19 pandemic in Australia, but then stabilised."

In Results, Temporal and cross-site validation, External validation at LRH in 2012-2021:

"PPV dropped from early 2017 until early 2019 before recovering. Sensitivity largely fell below the ***test*** *set estimate until mid-2015 and then declines again in the second half of 2020, continuing to decrease thereafter."*

7 Verification of the Effect of Text Standardization

It is mentioned that the vocabulary sharing ratio has increased to 75% after text standardization, but the necessity of this component has not been verified by ablation experiments (such as comparing the model performance difference before and after standardization). It is suggested to supplement the model expression under the condition of "no standardization" (such as PR AUC and PPV) to quantify the contribution of text standardization to the final result.

Response

Thank you for the great suggestion. We have run the experiments and added a section describing the results and showing text normalisation is indeed necessary.

In Methods, AI system for self-harm detection, Additional experiments:

"We also conducted an ablation study to evaluate the extent to which text normalisation improved downstream classification. We followed the same steps of the model development process as above,

*except this time using the raw triage notes from the **development** set. The best PR AUC was 0.81 (± 0.01), significantly lower than for normalised triage notes (Table S1)."*

8 Specific analysis of the difference of feature distribution

Fig. 3 shows that the Jensen-Shannon divergence of the feature distribution of the external verification set and the training set is large, but it does not specify which key features have the most significant distribution changes (for example, whether the frequency of keywords "excess" and "cut" related to self-injury is reduced in LRH records). It is suggested to list the top five features with the greatest difference, and analyze their influence on the decision boundary of the classifier (for example, whether the frequency difference of a feature in the training set and the verification set leads to the misjudgment of the model).

Response

Thank you for the great suggestion on how to further this analysis.

- We have examined the top keywords contributing to JS divergence.
- We have supplemented the results with analysis of SHAP values and the top keywords for self-harm classification within each dataset.

In Results, Corpus comparison analysis:

"To verify the portability of the AI system, we examined the distribution of tokens used as classifier inputs after text normalisation had been applied. As described above, the classification model was an ensemble of three base learners. Each base learner selected a set of features, adding up to 1931 unique features across the three sets. To assess their impact on model generalisation, we conducted two complementary analyses: (1) JS divergence analysis to quantify lexical distribution shift across datasets; (2) SHAP feature importance analysis to examine how model reliance on predictive features differs across corpora.

*Figure 5 illustrates that while a sufficient number of selected tokens persisted in the notes, their distribution varied. In the **prospective validation** set, the divergence increased sharply in early 2020. The **external validation** set shows considerable divergence from the **development** set throughout the entire 10-year period. Consistently, the TF-IDF representation shows higher JS divergence values compared to the count representation. This suggests differences are more pronounced in relative term importance than raw frequency alone.*

*Table 3 summarises the frequency of the top 10 most divergent features across quarters using TF-IDF representations. The **external validation** set revealed a stronger contribution from medication-related and substance-use terms like **"panadol"** (an Australian brand name for paracetamol), **"etoh"** (clinical abbreviation for alcohol), and **"mg"** (unit).*

*The top 10 features with the highest average global importance within each dataset are shown in Figure 6A. These features predominantly consist of clinical terms like **"pain"**, **"sob"** (abbreviation for shortness of breath), **"onset"**, and **"gcs"** (Glasgow Coma Scale). Within each dataset, the RMH data exhibited remarkably similar importance patterns, indicating internal consistency. The **external validation** set shared many of these features, but their relative rankings varied noticeably.*

*Finally, we calculated the difference in feature importance between the **development** set and each evaluation dataset and ranked features by the magnitude of this difference (Table S3). The greatest variation was observed between the **development** and **external validation** sets, with higher absolute*

*differences for terms related to medication intake, e.g., "x" (multiplier), **"taken", "mg", "took"**. These shifts in feature importance align with the observed lexical divergence in the JS analysis.*

Taken together, these observations reveal that in regional settings, both general complaints and self-harm presentations are more likely to involve medication ingestion. Furthermore, the results indicate self-poisoning, rather than self-injury, may be the more common primary mechanism of self-harm."

9 Practical guidance for model retraining

The discussion mentioned that LRH needs to retrain the classifier locally, but did not evaluate the minimum sample size or labeling cost required for retraining (such as how many manually labeled self-injury cases are needed to achieve the performance of the original model). It is suggested to supplement the preliminary experiments of "small sample learning" or "transfer learning" (such as the performance improvement after fine-tuning the model with 100/500 labeled data of LRH) to enhance the guiding significance for practical application.

Response

Thank you for advising on further experiments. We have run two sets of experiments:

1. Training the model on the RMH development set incrementally adding LRH data.
2. Training the model solely on LRH data.

In Methods, AI system for self-harm detection, External validation at LRH in 2012-2021:

"We investigated whether incorporating LRH data during training could remedy the diminished performance. We applied the original text normalisation to LRH triage notes from 2012 to 2017.

*First, we incrementally added one year's worth of LRH data to the **development** set and retrained the model. The best cross-validation PR AUC of 0.81 (± 0.01) was achieved when all six years of LRH data were combined with the original **development** set (Table S2).*

*Next, we removed the **development** set entirely and trained the model solely on LRH data. Again, only after incorporating all data from 2012 to 2017 did the model score a PR AUC of 0.85 (± 0.02). These experiments highlight the need for a substantial amount of local data to match performance at the development site, estimating 6,320 person-hours to gather six years' worth of data at LRH."*

10 Detailed description of data accessibility

The data availability statement mentions that "data can't be shared publicly, so it needs to be applied to the ethics committee", but it doesn't explain the application process (such as whether it needs institutional cooperation and ethical review time) or the data format (such as whether it needs to be unmarked or not, and field details). It is suggested to supplement the specific steps of data acquisition (such as the way of contacting Melbourne Health HREC and the requirements of application materials) to improve the verifiability of the study.

Response

We have added a Data Availability statement at the end of the manuscript.

In Data availability:

"This is highly sensitive clinical data from real patients, and access is restricted to bona fide research purposes. Please contact the corresponding author for support. An application for ethics and

governance approval must be made to the Royal Melbourne Hospital Human Research Ethics Committee (https://www.thermh.org.au/research/office-for-research/ethics/about-the-rmh-hrec)"

11 the distinction between self-injury and suicidal ideation

In this paper, "self-injury" and "suicidal ideation" are used alternately, but it is not clear whether the model distinguishes them (for example, whether suicidal ideation is marked as negative in training set). Since the goal of the model is to detect self-injury, it is necessary to explain the impact of suicide ideation cases on performance (for example, whether there is a case of mistaken classification of suicide ideation as self-injury, resulting in a decrease in PPV). It is suggested to supplement the confusion matrix to show the misclassification rate of self-injury and suicidal ideation.

Response

Thank you for pointing out the need to clarify the relationship between self-harm and suicidal ideation.

- We have added a definition of suicidal ideation.
- We have clarified how data were labelled and how the labels were grouped for modelling.
- We have added confusion matrices showing suicidal ideation cases are sometimes picked up by the model as positive for self-harm.

In Introduction:

"Self-harm is defined as any act of self-injury and/or self-poisoning irrespective of the degree of suicidal intent and/or other motivations [6]. In contrast, suicidal ideation refers to having suicidal thoughts or intention to act on those thoughts without current actions [7]."

In Methods, Data annotation:

"The textual component of each ED presentation was labelled as self-harm, suicidal ideation, or neither."

In Methods, Classification task:

"We trained the model to detect ED triage notes annotated as positive for self-harm; all notes negative for self-harm, including those positive for suicidal ideation, formed the negative class."

In Results, Model evaluation:

"We also investigated the confusion between self-harm and suicidal ideation. Out of the 227 false-positive notes, 53 were labelled as positive for suicidal ideation by annotators. This suggests approximately 23% of notes classified as self-harm may instead be positive for suicidal ideation (Figure S2f)."

Discussion on the feasibility of clinical application

Conclusion It is mentioned that the model can be used for "public health and potential clinical use" in RMH, but the specific scenarios of clinical application (real time diagnosis reminder, technical challenges of electronic health record integration) and the acceptance of clinicians (such as whether user surveys have been conducted) are not discussed. It is suggested to supplement the preliminary plan of clinical transformation (such as the pilot study design in cooperation with RMH) or demand (such as the response time of the model should be less than 1 second).

Response

We have outlined some of the next steps required to enable the clinical use of the tool.

In Conclusion:

"To enable its clinical use, a stronger ability to discern self-harm cases is required, particularly given the occasional confusion with suicidal ideation. Furthermore, analysis of the current workflow, the design of the optimal prediction delivery method, and considerations for the management of erroneous results are essential for clinical implementation of the system."

13 proofreading of language and format

There are grammatical errors or spelling problems in some expressions (for example, "reseacrh" should be "research"), and the terminology should be more consistent (for example, "text normalization" and "text normalization"). It is suggested to read the full text to ensure that the technical terms (such as "triage notes"“PR AUC" Prauc ") are used uniformly, and the chart titles and reference formats meet the requirements of the journal.

Response

Thank you and apologies for this. We have proofread the paper and supplementary materials and unified the terminology.

Reviewer #2:

This manuscript tackles an important question in clinical AI: whether natural-language machine learning models built on emergency triage notes can remain accurate across different hospitals and time periods. The authors evaluate a self-harm detection model using both new prospective data and data from a regional hospital, making the study timely and relevant to ongoing discussions about dataset shift and real-world AI performance. The paper is well written and well situated within current literature, and it provides valuable insight into the challenges of deploying NLP tools in diverse clinical environments.

There are a few notable strengths. The topic has strong clinical and public-health significance, and the study benefits from a robust design that includes prospective and external validation—still uncommon in medical AI. The authors provide transparent descriptions of their preprocessing and modelling approach and appropriately use PR-AUC for this imbalanced classification task. The discussion is well contextualised and highlights the broader implications for AI reliability and equity across regional health settings.

However, key improvements are needed to enhance clarity, methodological rigour, and interpretability. The largest gap relates to labelling: it is unclear how the external hospital's data were annotated, who performed the annotations, or whether definitions aligned with those used at the development site. Performance metrics are reported without confidence intervals, limiting the reader's ability to assess statistical significance. The manuscript would also benefit from a more detailed analysis of *why* performance degraded at the external site, including example misclassifications and qualitative differences in triage language. Vocabulary and divergence analyses are presented but require deeper interpretation and clearer visualisation. Threshold selection could be better justified, particularly in the context of recalibrating for a new site. Finally, the authors should consider discussing subgroup or fairness-related performance, given differences in patient populations.

Minor areas for improvement include streamlining the Methods section, clarifying the rationale for the selected feature set, improving figure readability, and correcting minor spelling inconsistencies.

Despite these issues, this is a strong and insightful manuscript. The conceptual separation of the NLP normalisation pipeline from the classifier is particularly commendable, and the temporal analysis is a meaningful contribution. With clearer reporting and a deeper examination of dataset shift, the paper could become a valuable reference for researchers developing portable AI systems in healthcare.

Response

Thank you for your positive and thoughtful feedback. We address the individual comments below but here is the summary of major changes in response to the key gaps you have highlighted:

- We have clarified that records from the two hospitals were labelled by the same team, over the same period of time, using the same definitions.
- We have provided additional information on the methodology, including feature and threshold selection, and re-calculated all metrics with confidence intervals for model evaluation.
- We have supplemented divergence analysis with differences in most frequent tokens and provided examples of misclassified notes along with the analysis of key contributing factors.
- We have reviewed figures, tables and thoroughly proofread the manuscript.

See key guidance and suggestions below:

Title:

Consider shortening for impact:

Response

We have reviewed the title to emphasise the impact of the study: Temporal and cross-site validation of an AI system for self-harm detection

Abstract – Background

- “Natural language–based machine learning models may not be portable…”

  → Clarify what aspect: semantics, syntax, local jargon, EHR structure.

- Add one sentence describing why self-harm detection matters (clinical significance).

Abstract – Methods

- Explicitly state whether the external validation dataset was manually annotated.
- Include 95% confidence intervals for performance measures.
- Place the PR-AUC results into clearer comparative format:
  - “Baseline RMH retrospective data: PR-AUC = 0.93 (CI…)”
  - “Temporal external data…” etc.
- Strengthen causal phrasing:
  - Instead of “suggests”, consider: “Performance degradation is primarily explained by linguistic domain shift and changes in triage documentation.”

Response

We have amended the abstract and the author’s summary as suggested above.

- We have clarified all data were manually annotated
- We have reported all metrics with 95% confidence intervals
- We have rephrased the results to draw direct comparison.
- We have amended the conclusion to make it sound more assertive

In Abstract:

“We leveraged emergency department data from two Australian hospitals, with free-text triage notes manually annotated for self-harm.”

“On the test set, the model achieved an area under the precision-recall curve (PR AUC) of 0.84 with a 95% confidence interval of [0.82, 0.86].”

“This performance remained stable at the development site with a PR AUC of 0.84, 95% CI [0.83, 0.85]. When applied in the regional context, the model’s ability to distinguish self-harm cases declined, resulting in an overall PR AUC of 0.78, 95% CI [0.77, 0.79].”

“At the metropolitan hospital, the AI system for self-harm detection maintained its epidemiological utility. At the regional hospital, the text normalisation process was effective, but performance was unstable primarily due to linguistic domain shift and differences in self-harm presentations.”

INTRODUCTION

Paragraph 1

- Add 1–2 citations for global burden of self-harm and ED presentation rates.
  - Sentence suggestion: "Self-harm accounts for approximately ___% of ED mental health presentations… [ref]"

Response

We have added global and Australian statistics to describe the global burden of suicide and self-harm.

In Introduction:

"Globally, over one million people die by suicide each year [1]. Suicide remains the leading cause of death for Australians aged 15 to 44, and in 2024 alone, suicide claimed 3,307 lives across all ages [2]."

Paragraph 2 (AI Context)

- Replace vague: "Real-world performance may differ substantially."

With:

"Real-world performance often degrades due to dataset shift in language, workflow, and patient demographics."

Response

We have strengthened the sentence as suggested.

In Background and Significance:

"In hospital settings, real-world performance often degrades due to various factors, ranging from differing patient demographics to site-specific protocols and data entry or management systems [16, 17]."

Paragraph 3 (Gaps)

- Clarify the three axes of dataset shift you assess: Temporal/Geographic/Workflow/EHR system change

Response

We have slightly rearranged the first two sections of the paper to improve the flow. We have broadly defined our aims at the end of the Introduction section moving the details down to the end of the Background & Significance section (see next point).

In Introduction:

"Triage notes manually annotated by suicide prevention experts revealed less than 3% of cases involved self-harm or suicidal ideation [15]. Despite such a data imbalance, our AI system demonstrated strong performance in detecting self-harm cases, though some confusion with suicidal ideation was noted. The system was deployed at Orygen, a youth mental health organisation, in Australia to support a self-harm monitoring system across the state of Victoria [6]. This study aims to confirm whether the system can be used prospectively at the development site and applied to other hospitals across the state."

Final Paragraph (Study Aims)

- Rephrase for clarity and structure:

"We aimed to assess (1) temporal robustness of the model during prospective validation at RMH, and (2) geographic portability at a regional hospital (LRH). We also examined whether differences in local linguistic patterns, documentation practices, or preprocessing vocabulary contributed to performance decline."

Response

Thank you for the suggestion, we have rephrased the study aims.

In Background & Significance:

"This study aims to evaluate the temporal robustness of the AI system at the development site and assess its cross-site portability. We explore how variations in local vocabulary and linguistic patterns influence differences in performance over time."

METHODS

- Add clearer demographic descriptions of RMH and LRH (population served, urban vs rural, proportion of Indigenous patients, etc.).
- Provide exact sample sizes with breakdowns:
  - Total triage notes
  - Total labelled cases
  - Prevalence at each site

Response

We have added the requested information:

- Descriptions of patient populations
- Number of collected, annotated, and analysed records
- Prevalence of self-harm at each site

In Results, Comparison of the two hospitals: Added population served and proportion of indigenous patients to Table 1.

In Methods, Data sources:

"In this study, we build on the 2012-2018 dataset compiled from ED presentations to RMH for the previous work [14], extending it to encompass the ten years 2012-2021. ED presentations to LRH spanned same period (2012-2021). Records were directly extracted from the respective hospital information systems. Data fields included age, gender, time and mode of arrival, and a triage comment based on the initial nursing assessment. The extracts contained 728,995 rows of data from RMH and 361,997 rows of data from LRH."

In Methods, Data annotation:

"All data from RMH and LRH were annotated by a team of trained researchers and experts in suicide prevention at Orygen, Australia."

In Results, Comparison of the two hospitals:

"The overall proportion of self-harm cases over the ten years at RMH vs. LRH was 1.4% and 1.7%, respectively. The proportion of suicidal ideation cases was only slightly higher, 1.5% vs. 1.9%. The number of ED presentations per quarter at each hospital and the trends in self-harm and suicidal ideation cases over time are shown in Figures 2a-b."

Major needed clarification.

Add a subsection titled "Annotation and Ground Truth Labelling":

- Who annotated?
- Training and calibration?
- Inter-rater agreement (κ score)?
- Consistency between RMH and LRH labels?

Response

Thank you for pointing out the need to clarify how data were annotated. We have added a subsection to the Methods section clarifying that all datasets were labelled by the same team, over the same period of time, using the same definitions. We have also quoted the inter-annotator rate from the previous study.

Methods, Data annotation:

"All data from RMH and LRH were annotated by a team of trained researchers and experts in suicide prevention at Orygen, Australia. The textual component of each ED presentation was labelled as self-harm, suicidal ideation, or neither. The annotators used the same definitions across the two sites and were blinded to other fields during the annotation process.

We estimated that annotation and subsequent audits take up to 20 person-hours per 1,000 notes. Due to the large volume of data, the notes were annotated by multiple annotators over a period of several years. As reported in our previous study [14], the inter-annotator agreement for self-harm, calculated as Cohen's kappa, was 0.91."

Normalization Pipeline

- List key steps in a figure or flowchart rather than a long paragraph.
- Provide examples of:
  - original triage text
  - normalized text output
  - common misspellings captured

Response

Thank you for the great suggestion.

- We have added a figure (Figure 3) visualising all steps of the AI system including the individual steps of text normalisation.
- We have supplemented each text normalisation step with several examples.
- We added an example of an original and normalised triage note.

In Methods, Text normalisation:

“Custom pre-processing

First, the text was converted to lowercase. Based on the development set, we developed a set of regular expressions to resolve commonly used shorthands, including “++ve” → “positive”, “r/ship” → “relationship”, “L)” → “left”.

Tokenisation

We used the domain-specific vocabulary to introduce whitespaces into composite tokens, for example, “warm/pink/dry” → “warm / pink / dry”, “20x5mg” → “20 x 5 mg”, while preserving time, dates, and vitals, e.g., not adding spaces to “2/7 ago”.

Spelling correction

We used the learned dictionary of misspellings to correct known misspellings, such as “aggitated” → “agitated”, “dizzyness” → “dizziness”, “intermittant” → “intermittent”.

Slang replacement

We replaced commonly used alternative names for relevant drugs, such as “fent” → “fentanyl”, “diaz” → “diazepam”.

Filtering

Lastly, we filtered out tokens that do not contain any letters, for example, removing “36.7” but retaining “t2dm”, a common notation for type 2 diabetes mellitus.”

In Results, AI system for self-harm detection

“The full AI system for self-harm detection in triage notes is presented in Figure 3.

Text normalisation

Passing a triage note through all steps of the text normalisation component resulted, for example, in the following transformation:

“Taken 20x5mg diaz. O/A GSC 15, drowsy but tearfull. PHx dep[ression. Multiple superficial lacs on L) arm from 2/7 ago” → “taken x mg diazepam o/a gcs drowsy but tearful phx depression multiple superficial lacs on left arm from ago”

Classifier Component

- Add rationale for choosing LightGBM vs alternatives.
- Clarify whether:
  - Term frequency or subword n-grams were included.
  - Stop words were removed.
  - Case sensitivity was preserved or removed.

Response

Thank you for pointing out the need to provide more information on the methodology. We have added an extensive section on the development of the self-harm classification model and specified the final set-up.

In Methods, Text normalisation, Custom pre-processing:

“First, the text was converted to lowercase.”

In Methods, Development of the classification model:

"We transformed text into its numerical representation using a term frequency-inverse document frequency (TF-IDF) vectoriser, which removed stop words and split tokens by whitespace. We retained features above a given percentile based on the chi-squared statistics. Other parameters of the vectoriser and the percentile value for feature selection were defined during hyperparameter tuning.

We compared four algorithms: multinomial Naive Bayes, Logistic Regression, Random Forest, and three implementations of the gradient boosting classifier (see Supplementary Materials, Model specifications). The three components were jointly optimised as a single model, including tuning hyperparameters of the vectoriser and the feature selector, to predict the probability of self-harm.

To select the best-performing model, we used a 5x3-fold nested cross-validation on the ***development*** *set. The inner loop tuned hyperparameters optimising for negative log loss. The outer loop compared models based on negative log loss, the area under the receiver operator characteristic curve (ROC AUC), and the precision-recall curve (PR AUC) of the positive class as secondary metrics.*

For final hyperparameter tuning, we used a 5-fold cross-validation on the ***development*** *set, optimising for PR AUC. This choice was guided by a significant class imbalance, where PR AUC is known to better reflect the model's classification ability on the minority class.*

To calibrate the model, we used a 5x3-fold nested cross-validation on the ***development*** *set. In the inner loop, the base estimator was fitted to the training data and calibrated on the testing data. The three fitted classifier and calibrator pairs were combined into an ensemble model, with the output being the average predicted probability of all pairs. We used the outer loop to compare uncalibrated model outputs with those calibrated using isotonic scaling. We examined the calibration curves and chose the version with the higher PR AUC.*

Finally, for each fold, we calculated the optimal probability threshold by maximising the F1 score – the point on the PR curve that best balances precision and recall. The average value was used as the final probability threshold.

The final classification model was retrained on the full ***development*** *set."*

In Results, AI system for self-harm detection, Self-harm classification model:

"Our self-harm classification model was developed on normalised triage notes from the ***development*** *set. Comparing different classifiers, the Light Gradient Boosting Machine (LGBM) classifier achieved the best results across all metrics. The best configuration involved calculating TF-IDF values for unigrams, bigrams, and trigrams, retaining the top 2% of features, and passing them into the LGBM classifier (for the full list of hyperparameters, see Supplementary Materials, Model specifications)."*

Evaluation Metrics

Add a sentence:

"All estimates were accompanied by 95% confidence intervals calculated using 1000 bootstrap iterations."

Response

We have added the suggested sentence and amended the reported metrics throughout the paper.

In Methods, Performance evaluation:

"For internal, prospective, and external validation, all estimates are reported as the mean with 95% confidence intervals derived from 1000 bootstrap iterations."

Comparison of Linguistic Differences

- Move technical formulas (JS divergence, TF-IDF cosine similarity) into Supplementary Materials.
- Provide interpretation guidance:
  - "JS divergence values closer to 0 indicate similarity, while values >0.2 indicate substantial shift."

Response

We have followed the suggestions, providing details on technical implementation in supplementary materials and adding a sentence to help interpret the results.

In Methods, Linguistic differences

"We compared distributions of selected features per quarter across each evaluation dataset with the entire ***development*** *set. We calculated pairwise Jensen-Shannon (JS) divergence using raw counts and TF-IDF representations to capture the difference in both raw lexicon usage and importance-weighted representation. JS divergence values closer to 0 indicate similarity, while values >0.2 indicate a substantial shift. We further analysed the per-feature contribution to explore if the divergence was concentrated on specific features."*

RESULTS

Descriptive Statistics

- Add a transparent cohort table .
  - Provide: median age, sex distribution, self-harm prevalence, missingness patterns

Response

We have added additional information to Table 1 summarising difference between patient populations, including the number of missing values for sex and arrival mode and the number of empty triage notes. We have reported on the prevalence of self-harm and suicidal ideation at each site.

In Results, Comparison of the two hospitals:

"The overall proportion of self-harm cases over the ten years at RMH vs. LRH was 1.4% and 1.7%, respectively. The proportion of suicidal ideation cases was only slightly higher, 1.5% vs. 1.9%. The number of ED presentations per quarter at each hospital and the trends in self-harm and suicidal ideation cases over time are shown in Figures 2a-b."

Temporal Results

- Clarify whether the drop in performance correlates with:
  - COVID-19 lockdown period/EHR migration/triage staff turnover
- Include:
  - "Figure X: PR curves with shaded confidence intervals."

Response

- Our revised results no longer demonstrate a strong correlation with COVID-19. We have added potential explanations where possible but given the current data we are unable to confirm them.

- We have created individual plots for each metric with the mean and confidence bands for each quarter.

In Results, Temporal and cross-site validation, Prospective validation at RMH in 2018-2021:

"PPV receded slightly in mid-2019 and sensitivity contracted around the first half of 2020, which coincided with the outbreak of the COVID-19 pandemic in Australia, but then stabilised."

In Results, Temporal and cross-site validation, External validation at LRH in 2012-2021:

"PPV dropped from early 2017 until early 2019 before recovering. Sensitivity largely fell below the ***test*** *set estimate until mid-2015 and then declines again in the second half of 2020, continuing to decrease thereafter."*

Geographic Validation

- Add sub-analysis exploring:
  - differences in top keywords
  - misclassified cases categorized by reason (e.g., ambiguous language, euphemisms)

Response

Thank you for the great suggestion on how to further this analysis.

- We have examined the top keywords contributing to JS divergence.
- We have supplemented the results with analysis of SHAP values and the top keywords for self-harm classification within each dataset.

In Results, Corpus comparison analysis:

"To verify the portability of the AI system, we examined the distribution of tokens used as classifier inputs after text normalisation had been applied. As described above, the classification model was an ensemble of three base learners. Each base learner selected a set of features, adding up to 1931 unique features across the three sets. To assess their impact on model generalisation, we conducted two complementary analyses: (1) JS divergence analysis to quantify lexical distribution shift across datasets; (2) SHAP feature importance analysis to examine how model reliance on predictive features differs across corpora.

Figure 5 illustrates that while a sufficient number of selected tokens persisted in the notes, their distribution varied. In the ***prospective validation*** *set, the divergence increased sharply in early 2020. The* ***external validation*** *set shows considerable divergence from the* ***development*** *set throughout the entire 10-year period. Consistently, the TF-IDF representation shows higher JS divergence values compared to the count representation. This suggests differences are more pronounced in relative term importance than raw frequency alone.*

Table 3 summarises the frequency of the top 10 most divergent features across quarters using TF-IDF representations. The ***external validation*** *set revealed a stronger contribution from medication-related and substance-use terms like* ***"panadol"*** *(an Australian brand name for paracetamol),* ***"etoh"*** *(clinical abbreviation for alcohol), and* ***"mg"*** *(unit).*

The top 10 features with the highest average global importance within each dataset are shown in Figure 6A. These features predominantly consist of clinical terms like ***"pain"***, ***"sob"*** *(abbreviation for shortness of breath),* ***"onset"***, *and* ***"gcs"*** *(Glasgow Coma Scale). Within each dataset, the RMH data*

exhibited remarkably similar importance patterns, indicating internal consistency. The ***external validation*** *set shared many of these features, but their relative rankings varied noticeably.*

Finally, we calculated the difference in feature importance between the ***development*** *set and each evaluation dataset and ranked features by the magnitude of this difference (Table S3). The greatest variation was observed between the* ***development*** *and* ***external validation*** *sets, with higher absolute differences for terms related to medication intake, e.g.,* ***"x"*** *(multiplier),* ***"taken"****,* ***"mg"****,* ***"took"****. These shifts in feature importance align with the observed lexical divergence in the JS analysis.*

Taken together, these observations reveal that in regional settings, both general complaints and self-harm presentations are more likely to involve medication ingestion. Furthermore, the results indicate self-poisoning, rather than self-injury, may be the more common primary mechanism of self-harm."

Error Analysis

- Provide five anonymised example triage notes highlighting:
  - true positive, false positive, false negative, mis-spellings not captured, domain-specific language

Response

We have included five examples of misclassified cases and examined the reasons for error.

In Results, Error analysis:

"Five anonymised examples are presented below, along with SHAP waterfall plots to illustrate the model's decision-making process.

Example of a true positive

Normalised triage note: *"pt taken oral overdose of x paracetamol and x diazepam pt reports he was intentionally trying to harm himself was in ed ago for same no vomiting no diarrhoea has some superficial self harm scratches on a arms"*

True label: Self-harm; predicted probability: 1.0; predicted label: Self-harm

This true positive shows the model performing as intended. Multiple explicit self-harm indicators (e.g., ***"intentionally"****,* ***"self harm"****,* ***"overdose"****) are correctly identified as the strongest positive contributors, pushing the prediction well above the threshold (f(x) = 12.009). The originally misspelled* ***"intentially"*** *was successfully corrected during text normalisation (Figure 6b).*

Example of a false positive

Normalised triage note: *"d/c from northern hospital yest post depressed suicidal thoughts and superficial lac 's to left wrist this am seen with knife by housemate no new lacerations flat affect hx bpd depression anxiety ptsd self harm"*

True label: Not self-harm; predicted probability: 0.99; predicted label: Self-harm

The note includes a rich psychiatric background: history of ***"self harm"****,* ***"superficial lac"*** *(laceration),* ***"depression"****, etc., which are all associated strongly with SHAP contribution. However, the phrase* ***"no new lacs"*** *indicates no active self-harm at this presentation, which was considered a negative label during annotation. This case shows the model lacks the ability of negation and temporal reasoning, resulting in a false positive prediction (Figure 6c).*

Example of a false negative

Normalised triage note: *"biba hours self harm approx cm laceration left inner wrist adipose tissue on view per av minimal blood loss dressed with av so not viewed left thumb altered sensation phx suicidal ideation increased social stressors vitals stable pain"*

True label: Self-harm; predicted probability: 0.3215; predicted label: Not self-harm

This false negative demonstrates an example close to the decision boundary. The predicted probability of 0.3215 falls short of the selected threshold of 0.3225. Despite clear signs of self-harm indicated in the note (e.g. ***"self harm"*** *+0.93), the strongly negative base value (E[f(x)] = -7.38) is only partially offset, resulting in a final score just under the threshold (Figure 6d).*

Example of a false negative due to misspellings

Normalised triage note: *"polupharmacy at hours agtiated on arrival heart rate sr on monitor direct to resus"*

True label: Self-harm; predicted probability: 0.0012; predicted label: Not self-harm

This false negative example shows how misspellings, ***"polupharmacy"*** *(polypharmacy) and* ***"agitated"*** *(agitated), can lead to misclassification. The model failed to capture the two characteristic features of self-harm, showing no positive contribution on the SHAP waterfall plot. Instead, the model relies on weak contextual features (****"hours"*** *+0.31,* ***"direct"*** *+0.21) that are insufficient to overcome the strongly negative base value (E[f(x)] = -7.38), resulting in a predicted probability of 0.0012 (Figure 6e).*

Example of a false negative due to non-specific terminology

Normalised triage note: *"onset dizziness stated taken aloe pain killers and has not eaten today recent d/c *** hosp not forthcoming with info phx autism"*

True label: Self-harm; predicted probability: 0.0002; predicted label: Not self-harm

This false negative shows how lay terminology, ***"taken a lot of pain killers"*** *used here to describe intentional overdose, causes model failure. Moreover,* ***"onset"*** *(-1.25) significantly contributes to the negative prediction, as it is a common term in general ED triage notes unrelated to self-harm (Figure 6f).*

Overall, these examples highlight that the model captures important self-harm-related features but is sensitive to lexical variation and standardisation of triage notes. Text normalisation and manual review of predictions close to the probability threshold could help improve prediction performance."

DISCUSSION

Opening Paragraph

- Remove repetition from the introduction about dataset shift.
- Consider reorganising the section:
    1. Summary of main findings
    2. Implications for NLP in healthcare
    3. Why performance declined in external setting
    4. What should happen next (calibration, retraining, small labelled dataset, etc.)

Consider adding subsections:

- Impact of EHR Migration (workflow shift)

- Sociolinguistic Variability Across Regions
- Role of Fine-Tuning with Small Local Dataset
- Fairness Considerations (e.g., Indigenous patient language patterns)

Strengthen policy relevance:

- "Health systems must consider ongoing monitoring, recalibration, and site-specific tuning before deploying NLP-based surveillance tools."

Response

We thank the reviewer for the generous input. We have reviewed the Discussion section, removing repetitions, reorganising the section following the suggested order and adding additional points on the costs of data annotation and the presence of mental health team on the ward. We have also incorporated the suggested sentence to strengthen our recommendation.

In Discussion:

"This study examines the temporal and cross-site portability of an NLP-based AI system for self-harm detection. We demonstrate that while the system retains its epidemiological utility, its accuracy decreases at the regional site. As discussed earlier, various environmental, technological, and organisational factors may continuously impact model performance. For example, while the presence of a 24/7 emergency mental health team at the RMH ED does not directly affect the quality of the notes—these are taken by nurses at the first encounter—there is an opportunity for training ED staff to better recognise and describe suicidal behaviours.

Our analysis of linguistic differences between hospitals reveals variations in self-harm presentations between metropolitan and regional populations. Features related to medication intake and substance consumption consistently rank higher in the regional hospital, suggesting self-poisoning as the more common method of self-harm.

A plethora of frameworks exist guiding the development and implementation of AI models in healthcare. The majority advise validating the candidate model in a new healthcare setting or on data from a different period [34]. This is usually sufficient to see that a model fails to generalise, as has been observed with the Epic Sepsis Model. However, a positive outcome of an external validation study does not guarantee the model's universal generalisability [35].

Given the temporal nature of the factors affecting the data distribution, assessing the performance over time is especially important and can yield insights into the model's underlying strengths and weaknesses. Recurring validation locally may be preferred over a one-off external validation [36]. Moreover, focus should be put on evaluating aspects relevant to the desired application rather than attempting to prove all-round generalisability [16]. A model yielding similar PPV and sensitivity may be valuable in the epidemiological context, even if its overall discriminatory ability is not appropriate for clinical use.

This study also highlights the importance of assessing regional variation. Most healthcare AI models are developed using data from major tertiary hospitals or research centres. Their portability to regional settings is limited not only by infrastructural and information systems aspects. Service availability, varying health literacy levels, and preference for community-based support can impact how patients interact with healthcare providers. In regional areas, patients may present with different risk factors or face unique socioeconomic challenges that influence their healthcare needs and outcomes.

Furthermore, the patterns of suicide-related behaviours might differ between urban and rural populations, affecting model performance. To deliver equitable care, AI models must be adaptable to these diverse contexts. Health systems must consider ongoing monitoring using application-specific metrics, recalibration, and tuning on local data before deploying NLP-based surveillance tools.

However, data annotation presents its own challenges. Appropriately trained personnel may be scarce in regional and remote areas. Conditions like self-harm and suicidal ideation are relatively subjective and often difficult to discern from the limited amount of information captured in nursing triage notes. Finally, in terms of human labour costs, collecting and annotating sufficient local data requires a significant time commitment.

Given these factors, we stress the importance of validating individual system components, comparing corpora to development data before annotation, and the move towards temporal evaluation of AI models."

CONCLUSION

Revise and consider:

- "Future work will evaluate lightweight retraining, rapid calibration using small labelled batches, and deployment workflows to ensure equitable self-harm detection across diverse ED settings."

Response

Thank you for the suggestion. We have amended the Conclusion as follows:

<u>In Conclusion:</u>

"We demonstrate that the AI system for self-harm detection maintains its utility at the original site when validated prospectively. Balanced PPV and sensitivity suggest the model could be used to reliably track the proportion of self-harm cases in the population as an operational epidemiological tool embedded in public health workflows. The AI tool has been deployed to support a state-wide self-harm monitoring system, providing an opportunity for ongoing validation and refinement.

Future work will focus on investigating the variations in self-harm presentations and exploring the use of more sophisticated AI methods to normalise data and extract discrete information from triage notes.

To enable its clinical use, a stronger ability to discern self-harm cases is required, particularly given the occasional confusion with suicidal ideation. Furthermore, analysis of the current workflow, the design of the optimal prediction delivery method, and considerations for the management of erroneous results are essential for clinical implementation of the system."

FIGURES / TABLES

Figures

- Ensure all axes are clearly labelled with metric and CI.
  - Improve readability using a consistent color palette, larger font, shading for confidence intervals

Response

We have unified and increased the font size and ensured a consistent colour palette. We have visualised confidence bands for all metrics.

Tables

- Consider adding a new Table 1 (Cohort Characteristics) and Table 2 (Model Performance) with 95% CI.

Response

We have reordered the results, provided additional information in Table 1 (Comparison of the two healthcare providers), and reported confidence intervals in Table 2 (Overall performance of the self-harm classification model).
