# Supplementary material for: Temporal and cross-site validation of an AI system for self-harm detection
Source: PLOS Digit Health. 2026 Sep 11;5(9):e0001667. doi: 10.1371/journal.pdig.0001667 (PMC13567808; doi:10.1371/journal.pdig.0001667)
Supplement: Supplementary file 2 [file pdig.0001667.s015.pdf]

Dear reviewers and the editorial team,

On behalf of all co-authors, I would like to thank you for the additional comments. We have carefully addressed the journal requirements and incorporated the reviewers' suggestions. We appreciate the time and effort you invested in reviewing our manuscript. Please let us know if there are any remaining concerns.

Our responses to all your comments are available on the next page.

Best wishes,

Vlada Rozova

Journal Requirements:

1. Please amend your detailed Financial Disclosure statement. This is published with the article. It must therefore be completed in full sentences and contain the exact wording you wish to be published.

    i. Please clarify all sources of financial support for your study. List the grants, grant numbers, and organizations that funded your study, including funding received from your institution. Please note that suppliers of material support, including research materials, should be recognized in the Acknowledgements section rather than in the Financial Disclosure.

    ii. State the initials, alongside each funding source, of each author to receive each grant. For example: "This work was supported by the National Institutes of Health (####### to AM; ###### to CJ) and the National Science Foundation (###### to AM)."

    iii. State what role the funders took in the study. If the funders had no role in your study, please state: "The funders had no role in study design, data collection and analysis, decision to publish, or preparation of the manuscript."

    iv. If any authors received a salary from any of your funders, please state which authors and which funders.

Response

Please find the amended Financial Disclosure statement below:

The establishment of the emergency department surveillance system received funds from the Victorian Department of Health, the William Buckland Foundation, and Future Generation Global (all to JR). Salaries were provided by the Medical Research Future Fund (MRFCRI000188 and MRFFRD000113 to VR), the National Health and Medical Research Council (#1177787 to KW; #2008460 to JR), and the University of Melbourne (Dame Kate Campbell Fellowship to KW; Dame Kate Campbell Fellowship to JR). The funders had no role in study design, data collection and analysis, decision to publish, or preparation of the manuscript.

2. We note that you have indicated that there are restrictions to data sharing for this study. For studies involving human research participant data or other sensitive data, we encourage authors to share de-identified or anonymized data. However, when data cannot be publicly shared for ethical reasons, we allow authors to make their data sets available upon request. For information on unacceptable data access restrictions, please see http://journals.plos.org/plosone/s/data-availability#loc-unacceptable-data-access-restrictions.

    Before we proceed with your manuscript, please address the following prompts:

    a) If there are ethical or legal restrictions on sharing a de-identified data set, please explain them in detail (e.g., data contain potentially identifying or sensitive patient information, data are owned by a third-party organization, etc.) and who has imposed them (e.g., a Research Ethics Committee or Institutional Review Board, etc.). Please also provide contact information for a data access committee, ethics committee, or other institutional body to which data requests may be sent.

    b) If there are no restrictions, please upload the minimal anonymized data set necessary to replicate your study findings to a stable, public repository and provide us with the relevant URLs, DOIs, or accession numbers. Please see http://www.bmj.com/content/340/bmj.c181.long for guidelines on how to de-identify and prepare clinical data for publication.

For a list of recommended repositories, please see https://journals.plos.org/plosone/s/recommended-repositories. You also have the option of uploading the data as Supporting Information files, but we would recommend depositing data directly to a data repository if possible.

Please update your Data Availability statement in the submission form accordingly.

Response

Please find the amended Data Availability statement below:

The dataset used in this study consists of emergency department triage notes from two Australian hospitals, the Royal Melbourne Hospital and Latrobe Regional Hospital. The free-text notes, whilst stripped of direct identifiers (e.g., patient names, Medicare numbers), retain features — including specific dates, clinical event descriptions, and references to named locations — that present a residual re-identification risk under the Australian Privacy Act 1988 (Cth) when considered alongside publicly available auxiliary data. This risk cannot be eliminated without destroying the clinical utility of the notes.

Ethics approval was granted under a waiver of individual patient consent by the Melbourne Health Human Research Ethics Committee (HREC; Approval No. 2017.342). Approval conditions restrict data access to named research team members and prohibit open or third-party release. Access is further governed by data sharing agreements with each participating hospital site.

Researchers seeking access to the data for legitimate research purposes should contact: Professor Jo Robinson, Orygen / Centre for Youth Mental Health, The University of Melbourne, jo.robinson@unimelb.edu.au

Requests will be assessed against the conditions of HREC Approval No. 2017.342 and applicable privacy legislation. Requests involving use cases beyond the scope of the original approval may require independent ethics review. A data sharing agreement with the relevant hospital site(s) may also be required.

3. Some material included in your submission may be copyrighted. According to PLOS's copyright policy, authors who use figures or other material (e.g., graphics, clipart, maps) from another author or copyright holder must demonstrate or obtain permission to publish this material under the Creative Commons Attribution 4.0 International (CC BY 4.0) License used by PLOS journals. Please closely review the details of PLOS's copyright requirements here: PLOS Licenses and Copyright. If you need to request permissions from a copyright holder, you may use PLOS's Copyright Content Permission form.

Please respond directly to this email or email the journal office and provide any known details concerning your material's license terms and permissions required for reuse, even if you have not yet obtained copyright permissions or are unsure of your material's copyright compatibility.

Potential Copyright Issues:

a. Figures 1 and 3: Please confirm whether you drew the images / clip-art within the figure panels by hand. If you did not draw the images, please provide (a) a link to the source of the images or icons and their license / terms of use; or (b) written permission from the copyright holder to publish the images or icons under our CC-BY 4.0 license. Alternatively, you may replace the images with open source alternatives. See these open source resources you may use to replace images / clip-art:

- https://commons.wikimedia.org
- https://openclipart.org/

Response

Figure 1 was created in Keynote using icons available within the app. Figure 3 was generated using FigureLabs software.

As per Jessica Thomas and her team, we have been advised that "prebuilt icons or content from the Keynote's app is not compatible with our publication license and will need to be removed or replaced." We have removed all icons from Figure 1.

We were also advised that "Figure 3 (Figurelabs) should be okay to proceed, please ensure that the source is clearly referenced in the figure legend." We have amended the caption to Figure 3 to reflect the source:

Figure 3. The AI system for self-harm detection. *The system has two components: text normalisation and a self-harm classification model. Text normalisation consists of custom pre-processing, tokenisation, spelling correction, slang replacement, and token filtering. The classification model is an ensemble of three base learners, each including a vectoriser, a feature selector, and a LGBM classifier. The figure was created using the FigureLabs illustrator.*

4. Please upload separate figure files in .tif or .eps format. Also, remove the figures from your manuscript file but keep the legends.

For more information about figure files please see our guidelines:

https://journals.plos.org/digitalhealth/s/figures
https://journals.plos.org/digitalhealth/s/figures#loc-file-requirements

Response

We have removed all figures from the manuscript file and uploaded them separately in .tif. Please let us know if the quality is not acceptable.

If the reviewer comments include a recommendation to cite specific previously published works, please review and evaluate these publications to determine whether they are relevant and should be cited. There is no requirement to cite these works unless the editor has indicated otherwise.

Reviewer #3:

This is an excellent, well-executed validation study that makes a timely and significant contribution to the field of clinical AI. The authors have performed a rigorous and comprehensive assessment of their previously developed NLP system for detecting self-harm in emergency department triage notes. The study robustly addresses the critical question of model portability across both time and different healthcare settings. The revisions made in response to the reviewers' comments are thorough and have substantially strengthened the manuscript's clarity, methodological rigour, and depth of analysis. The work is now suitable for publication in its current form.

- The following minus notes would be good to address：

The observed confusion between self-harm and suicidal ideation in model predictions is an interesting finding. Further brief reflection on how this might be managed in an operational public health surveillance or clinical decision-support context could be a valuable addition to future work.

- The logical next steps for clinical implementation (e.g., a feasibility pilot within the electronic health record system) are alluded to. A slightly more concrete outline of this translational pathway could further guide the research community.

Response

The authors would like to thank the reviewer for the favourable review and suggestions for additional reflection points. We have added the following two paragraphs:

In Conclusion:

As indicated by the error analysis, when searching for self-harm presentations, end-users should pay attention to history of self-harm and consider potential confusion with suicidal ideation. Users may also choose to review cases with predicted probabilities just below the threshold. While manual review is still required, the algorithm dramatically reduces the amount of work, with less than 3% of notes assigned the probability above 0.1.

To enable its clinical use, a stronger ability to discern self-harm cases is required, particularly given the occasional confusion with suicidal ideation. Furthermore, analysis of the current workflow, the design of the optimal prediction delivery method, and considerations for the management of erroneous results are essential for clinical implementation of the system. A silent trial followed by a single-centre randomised controlled trial would be the initial steps in exploring the various factors involved in bringing an AI-based system into clinical practice.

Reviewer 4

This manuscript presents a valuable study on self-harm surveillance. Building on prior work, the authors rigorously assessed the portability and temporal robustness of the self-harm classification model through prospective and external validation. The findings indicate that linguistic domain shift and differing presentations of self-harm contribute to a notable decline in positive predictive value (PPV). The study supports the development of a national suicide prevention system and offers valuable enlightening insights for peers carrying out other relevant similar researches.

The methods and technical approach are sound. The performance metrics are satisfactory under the evaluated conditions. Minor grammatical or stylistic issues do not detract from the manuscript.

I recommend discussing local linguistic patterns and differences in self-harm presentations, which may explain the drop in PPV. Potential dataset biases, including patient demographics, regional language usage, or living conventions, could be elaborated. Such analysis would clarify factors underlying reduced model performance, and is seems more important for readers from diverse national and cultural contexts.

Response

The authors would like to thank the reviewer for the favourable review and suggestions on how to improve the readability of the paper. We have proofread the manuscript once again for grammatical errors.

We conducted in-depth analysis to quantify the lexical distribution shift across corpora and the differences in selected features. We have added a pointer to that section of the manuscript. Our comparison of demographics (Table 1) is based on nationally reported statistics and publicly available data. More concrete insights into the differences in regional language and socio-economic factors would require linking the ED triage notes to these data, which is beyond the scope of this study.

In Results, Temporal and cross-site validation, External validation at LRH in 2012-2021:

Falling short of the original performance, these results indicate critical differences in underlying data. In the following sections, we examine whether these differences are inherent to the corpora or are an unintended effect of text normalisation.

Reviewer 5

This is a revised manuscript that addresses a practical and clinically meaningful question: Whether AI self-harm detection model is generalizable across hospital sites. The authors have addressed most of the earlier concerns from previous reviewers and I can see that the revised version is substantially improved. Here, I have several comments that may further help improve the integrity and quality of the work.

1. Please clarify whether the clinical labels distinguish "current self-harm presentation" from "history of self-harm." The false-positive example suggests that the model may classify a note as positive when self-harm is mentioned only as history. It would be helpful to state clearly in the annotation section that the positive label refers to self-harm related to the current ED presentation, not past history alone.
2. The part of error analysis is very useful, as it points out a few cases that we still require manual review. I'd mention this and discuss a bit more in the limitation paragraph in Discussion section.
3. The added confidence bands and feature-importance plots are useful, but these figures may become visually dense. Please make sure that the axis labels, legends, and font sizes remain readable and consistent in the final journal format (e.g. make Figure 6BCDEF font sizes the same as in Panel A).
4. Also some y-limit axes could be zoomed in, otherwise it is very hard to eyeball the differences across different sets. For example, Figure 4 G and H.
5. I found the thresholding strategy a little hard to follow. The manuscript says the final probability threshold was selected by maximizing F1 score, and later gives an example where a case with predicted probability 0.3215 was classified as negative because it fell just below the threshold of 0.3225. However, it is not clear to me whether this threshold was chosen only once using the RMH development data and then applied unchanged to the RMH prospective and LRH external validation datasets, or whether it was adjusted for LRH. I suggest the authors state this explicitly in the Methods.

Response

The authors would like to thank the reviewer for the practical suggestions on how to improve the quality of the paper.

1. We have clarified that positive self-harm label include only current self-harm episodes and not historical self-harm.

<u>In Methods, Data Annotation:</u>

Only presentations linked to a current episode of self-harm or suicidal ideation were labelled positive. A note indicating a history of self-harm or suicidal ideation alone would be annotated as negative.

2. We have described how our end-users use the tool based on the known sources of errors.

<u>In Conclusion:</u>

As indicated by the error analysis, when searching for self-harm presentations, end-users should pay attention to the history of self-harm and consider potential confusion with suicidal ideation. Users may also choose to review cases with predicted probabilities just below the threshold. While manual review is still required, the algorithm dramatically reduces the amount of work, with less than 3% of notes assigned a probability above 0.1.

3. We have increased all font sizes (titles, axis ticks and labels, legends) and made sure they are consistent across all figures.
4. Thank you for the suggestion. The y axes in Figures 4G and 4H now range from 0.9 to 1.0, so the changes in specificity can be better seen. We note this in the caption.

Figure 4. Performance of the AI system for self-harm detection over time. *PR AUC (a-b), PPV (c-d), sensitivity (e-f), and specificity (g-h; note the altered y-axis scale) on the* ***prospective validation*** *(left) and* ***external validation*** *(right) datasets. Dashed lines indicate estimates calculated over the full set of triage notes as reported in Table 2. Solid lines show mean scores with 95% confidence bands over time, where triage notes were grouped into quarters based on arrival dates.*

5. We see the probability threshold as another model hyperparameter. As such, once tuned, it is applied unchanged during validation. We have clarified this in the Methods section.

In Methods, Development of the Classification Model:

The threshold was treated as a model hyperparameter and once tuned, remained unchanged during both prospective and external model validation.
