# Supplementary material for: Temporal and cross-site validation of an AI system for self-harm detection
Source: PLOS Digit Health. 2026 Sep 11;5(9):e0001667. doi: 10.1371/journal.pdig.0001667 (PMC13567808; doi:10.1371/journal.pdig.0001667)
Supplement: Supplementary file 3 [file pdig.0001667.s009.docx]

## Learning a domain-specific vocabulary

We iteratively constructed the vocabulary by leveraging several resources:

- Word lists from the SCOWL database [1] to include both general English words and Australian variants.
- A list of terms from the SNOMED CT-AU Australian ED reference set [2], for ED-specific terminology.
- Lists of terms from the SNOMED CT-AU medicinal and trade product reference sets [3, 4], for medication names.
- A comprehensive list of clinical abbreviations produced by the New South Wales Ministry of Health, Australia [5].
- A list of places in Victoria, Australia, scraped from a local government website.
- A manually curated list of the most frequently occurring words in the *development* set that were not covered by any of the lists above (for example, names of local hospitals and their abbreviations, such as “RMH”).

In the final step, we filtered out words and abbreviations that did not appear in the *development* set. Given the relatively large number of triage notes (nearly 320,000), we reasoned that words not present in the *development* set are either very rare and do not carry important information or are not relevant to the ED context. The resulting vocabulary consisted of 20,115 words. Here, a word is a token that contains at least one letter: punctuation and numerical measurements are ignored, but “t2dm”, a common notation for type 2 diabetes mellitus, is considered to be a valid word.

### References

1. SCOWL (Spell Checker Oriented Word Lists) and Friends.
2. Agency, A.D.H., Australian emergency department reference set. National Clinical Terminology Service.
3. Agency, A.D.H., Medicinal product reference set. National Clinical Terminology Service.
4. Agency, A.D.H., Trade product reference set. National Clinical Terminology Service.
5. District, S.E.S.L.H., Clinical Abbreviations List, N.M.o. Health, Editor
