# Supplementary material for: Temporal and cross-site validation of an AI system for self-harm detection
Source: PLOS Digit Health. 2026 Sep 11;5(9):e0001667. doi: 10.1371/journal.pdig.0001667 (PMC13567808; doi:10.1371/journal.pdig.0001667)
Supplement: Supplementary file 4 [file pdig.0001667.s010.docx]

## Learning a dictionary of misspellings

To correct misspellings, we used the Python library pyspellchecker. The spell checker was initialised with the domain-specific vocabulary. For each out-of-vocabulary (OOV) word, a set of candidates from the vocabulary was selected based on the Levenshtein distance. For each OOV word in the *development* set, we recorded the likeliest candidate, thus compiling a dictionary of misspellings. For OOV words that occurred in the development set more than 100 times, we manually reviewed and fixed the results of spelling correction.
