# Supplementary material for: Temporal and cross-site validation of an AI system for self-harm detection
Source: PLOS Digit Health. 2026 Sep 11;5(9):e0001667. doi: 10.1371/journal.pdig.0001667 (PMC13567808; doi:10.1371/journal.pdig.0001667)
Supplement: Supplementary file 5 [file pdig.0001667.s011.docx]

Model specifications

The full list of stop words can be found in the GitHub repository in self_harm_triage_notes/dev_utils.py.

For the gradient boosting machines, we tested three implementations: scikit-learn native GradientBoostingClassifier(), XGBClassifier() from the xgboost library, and LGBMClassifier() from the lightgbm library.

When tuning hyperparameters of the LGBM classifier, we allowed for the following ranges of parameters:

```
param_grid = {
    'tfidfvectorizer__ngram_range': ((1, 1), (1, 2), (1, 3), (2, 2)),
    'tfidfvectorizer__max_df': (0.8, 0.9, 1.0),
    'tfidfvectorizer__min_df': (10, 20),
    'tfidfvectorizer__sublinear_tf': (True, False),
    'selectpercentile__percentile': uniform(0.1, 3.0), # or 15.0 to allow for more features to be selected
    'lgbmclassifier__num_leaves': [5, 10, 30, 50, 100],
    'lgbmclassifier__max_depth': [-1, 3, 4, 5, 6, 7, 10],
    'lgbmclassifier__learning_rate': [0.1],
    'lgbmclassifier__n_estimators': [300, 400, 500],
    'lgbmclassifier__min_child_samples': range(1, 50),
    'lgbmclassifier__subsample': uniform(0.5, 1.0),
    'lgbmclassifier__colsample_bytree': uniform(0.5, 1.0),
    'lgbmclassifier__reg_alpha': uniform(0.0, 1.0),
    'lgbmclassifier__reg_lambda': uniform(0.0, 1.0),
    'lgbmclassifier__is_unbalance': [True, False],
}
```
