# Supplementary material for: Temporal and cross-site validation of an AI system for self-harm detection
Source: PLOS Digit Health. 2026 Sep 11;5(9):e0001667. doi: 10.1371/journal.pdig.0001667 (PMC13567808; doi:10.1371/journal.pdig.0001667)
Supplement: Supplementary file 6 [file pdig.0001667.s012.docx]

Error analysis

Waterfall plots display the contribution of individual features to the final prediction. Starting from this baseline, we accumulate feature contributions to arrive at the final log-odds score f(x). The baseline, E[f(x)], reflects the model's prior towards the negative class due to class imbalance. Note that f(x) is not directly interpretable as a probability. It represents the raw log-odds output of the underlying tree model. The final probability score is obtained by transforming f(x) through the sigmoid function, and then averaging across the base learners in the ensemble.
