# Supplementary material for: Temporal and cross-site validation of an AI system for self-harm detection
Source: PLOS Digit Health. 2026 Sep 11;5(9):e0001667. doi: 10.1371/journal.pdig.0001667 (PMC13567808; doi:10.1371/journal.pdig.0001667)
Supplement: Supplementary file 7 [file pdig.0001667.s013.docx]

Model Hyperparameters

The final self-harm classification model was an ensemble of three base learners fitted with the following hyperparameters:

```
vectorizer = TfidfVectorizer(stop_words=get_stopwords(), token_pattern=r'\S+')
selector = SelectPercentile(score_func=chi2)
clf = LGBMClassifier(force_row_wise=True, random_state=42, verbose=-1)
pipeline = make_pipeline(vectorizer, selector, clf)
params = {'lgbmclassifier__colsample_bytree': 0.7101410610866284,
          'lgbmclassifier__learning_rate': 0.1,
          'lgbmclassifier__max_depth': 6,
          'lgbmclassifier__min_child_samples': 4,
          'lgbmclassifier__n_estimators': 500,
          'lgbmclassifier__num_leaves': 50,
          'lgbmclassifier__reg_alpha': 0.778976006508152,
          'lgbmclassifier__reg_lambda': 0.5801504076624308,
          'lgbmclassifier__subsample': 0.812912541609473,
          'selectpercentile__percentile': 1.9840691514980384,
          'tfidfvectorizer__max_df': 0.9,
          'tfidfvectorizer__min_df': 10,
          'tfidfvectorizer__ngram_range': (1, 3),
          'tfidfvectorizer__sublinear_tf': True}
tuned_model = pipeline.set_params(**params)
```
