# Supplementary material for: Temporal and cross-site validation of an AI system for self-harm detection
Source: PLOS Digit Health. 2026 Sep 11;5(9):e0001667. doi: 10.1371/journal.pdig.0001667 (PMC13567808; doi:10.1371/journal.pdig.0001667)
Supplement: Peer review history

# Peer review: Decision Letter 0

**DOI:** 10.1371/journal.pdig.0001667.r001

**Authors:** Laura Sbaffi, Phat Kim Huynh

17 Dec 2025

Response to ReviewersRevised Manuscript with Track ChangesManuscript**Journal Requirements:** **Additional Editor Comments (if provided):** **Reviewers' Comments:**

**Comments to the Author**

1. Does this manuscript meet PLOS Digital Health’s publication criteria?

Reviewer #1: Yes

Reviewer #2: Yes

**********

2. Has the statistical analysis been performed appropriately and rigorously?

Reviewer #1: Yes

Reviewer #2: Yes

**********

3. Have the authors made all data underlying the findings in their manuscript fully available (please refer to the Data Availability Statement at the start of the manuscript PDF file)?

The PLOS Data policy

Reviewer #1: Yes

Reviewer #2: No

**********

4. Is the manuscript presented in an intelligible fashion and written in standard English?

Reviewer #1: No

Reviewer #2: Yes

**********

Reviewer #1: 1 The depth and necessity of the research background

Although the importance of self-injury monitoring in suicide prevention is mentioned in this paper, it does not fully link the shortcomings of traditional diagnostic coding with the advantages of NLP model. It is suggested to supplement the specific limitations of traditional methods (such as ICD coding) in self-injury detection (such as the rate of missing report and delay), and quote the latest research data (such as the empirical evidence of missing report of self-injury by diagnostic coding in recent years) to strengthen the necessity of this study.

2 Reproducibility of model development details

The method part mentions that the model uses 644 selected features, but the specific method of feature selection (such as feature importance, chi-square test or L1 regularization) is not clear. The criteria of feature selection (such as threshold setting and exclusion criteria) and verification process (such as how to determine the optimal number of features in cross-verification) need to be supplemented to ensure the reproducibility of the method.

3 transparency and basis of data preprocessing

The reason for excluding patients under 8 years old is "it is difficult to determine the intention of self-injury", but the specific proportion of self-injury events in this age group (such as the proportion of patients under 8 years old in the original data and the number of self-injury cases) or related research support is not provided. It is suggested to supplement the literature basis of exclusion criteria (such as clinical guidelines for the evaluation of children's self-injury intention) and discuss the potential impact of this exclusion on the generalization of the model (such as whether to limit the application of the model in pediatric scenes).

4. Selection basis of external verification hospitals

Regional hospital (LRH) was selected for external verification, but the key differences between LRH and the original hospital (RMH) in medical practice and recording habits (such as whether there is an emergency psychiatric team and the standardization of triage records) were not explained in detail. Table 2 compares some demographic information, but it is necessary to further discuss how these differences (such as whether the non-psychiatric team of LRH leads to a more vague description of self-injury in triage records) may affect the model performance.

5 Selection and interpretation of performance indicators

The study selected PR AUC, PPV, sensitivity and other indicators, but did not clearly explain why these indicators were selected (such as the reason why PR AUC is better than ROC AUC when the data is unbalanced). It is suggested to supplement the theoretical basis for the selection of indicators (for example, "the proportion of self-injury cases is less than 2%, and PR AUC can better reflect the performance of the model in a few categories"), and explain the practical significance of the specificity close to 1 (for example, the misjudgment rate of non-self-injury cases is extremely low).

6 mechanism analysis of time trend

Figure 2 shows that the performance of LRH decreased (sensitivity decreased to 0.68) after Q2, 2020, which was attributed to COVID-19 pandemic, but no specific evidence was provided (for example, the contents of triage records changed during the pandemic: was the record shorter and the terminology less standardized due to emergency? Has the type of self-injury changed? ）。 It is suggested to combine the adjustment of medical practice during the pandemic (such as remote triage and recording priority changes) and analyze its influence on text characteristics and model input.

7 Verification of the Effect of Text Standardization

It is mentioned that the vocabulary sharing ratio has increased to 75% after text standardization, but the necessity of this component has not been verified by ablation experiments (such as comparing the model performance difference before and after standardization). It is suggested to supplement the model expression under the condition of "no standardization" (such as PR AUC and PPV) to quantify the contribution of text standardization to the final result.

8 Specific analysis of the difference of feature distribution

Fig. 3 shows that the Jensen-Shannon divergence of the feature distribution of the external verification set and the training set is large, but it does not specify which key features have the most significant distribution changes (for example, whether the frequency of keywords "excess" and "cut" related to self-injury is reduced in LRH records). It is suggested to list the top five features with the greatest difference, and analyze their influence on the decision boundary of the classifier (for example, whether the frequency difference of a feature in the training set and the verification set leads to the misjudgment of the model).

9 Practical guidance for model retraining

The discussion mentioned that LRH needs to retrain the classifier locally, but did not evaluate the minimum sample size or labeling cost required for retraining (such as how many manually labeled self-injury cases are needed to achieve the performance of the original model). It is suggested to supplement the preliminary experiments of "small sample learning" or "transfer learning" (such as the performance improvement after fine-tuning the model with 100/500 labeled data of LRH) to enhance the guiding significance for practical application.

10 Detailed description of data accessibility

The data availability statement mentions that "data can't be shared publicly, so it needs to be applied to the ethics committee", but it doesn't explain the application process (such as whether it needs institutional cooperation and ethical review time) or the data format (such as whether it needs to be unmarked or not, and field details). It is suggested to supplement the specific steps of data acquisition (such as the way of contacting Melbourne Health HREC and the requirements of application materials) to improve the verifiability of the study.

11 the distinction between self-injury and suicidal ideation

In this paper, "self-injury" and "suicidal ideation" are used alternately, but it is not clear whether the model distinguishes them (for example, whether suicidal ideation is marked as negative in training set). Since the goal of the model is to detect self-injury, it is necessary to explain the impact of suicide ideation cases on performance (for example, whether there is a case of mistaken classification of suicide ideation as self-injury, resulting in a decrease in PPV). It is suggested to supplement the confusion matrix to show the misclassification rate of self-injury and suicidal ideation.

Discussion on the feasibility of clinical application

Conclusion It is mentioned that the model can be used for "public health and potential clinical use" in RMH, but the specific scenarios of clinical application (real time diagnosis reminder, technical challenges of electronic health record integration) and the acceptance of clinicians (such as whether user surveys have been conducted) are not discussed. It is suggested to supplement the preliminary plan of clinical transformation (such as the pilot study design in cooperation with RMH) or demand (such as the response time of the model should be less than 1 second).

13 proofreading of language and format

There are grammatical errors or spelling problems in some expressions (for example, "reseacrh" should be "research"), and the terminology should be more consistent (for example, "text normalization" and "text normalization"). It is suggested to read the full text to ensure that the technical terms (such as "triage notes”“PR AUC" Prauc ") are used uniformly, and the chart titles and reference formats meet the requirements of the journal.

Reviewer #2: This manuscript tackles an important question in clinical AI: whether natural-language machine learning models built on emergency triage notes can remain accurate across different hospitals and time periods. The authors evaluate a self-harm detection model using both new prospective data and data from a regional hospital, making the study timely and relevant to ongoing discussions about dataset shift and real-world AI performance. The paper is well written and well situated within current literature, and it provides valuable insight into the challenges of deploying NLP tools in diverse clinical environments.

There are a few notable strengths. The topic has strong clinical and public-health significance, and the study benefits from a robust design that includes prospective and external validation—still uncommon in medical AI. The authors provide transparent descriptions of their preprocessing and modelling approach and appropriately use PR-AUC for this imbalanced classification task. The discussion is well contextualised and highlights the broader implications for AI reliability and equity across regional health settings.

However, key improvements are needed to enhance clarity, methodological rigour, and interpretability. The largest gap relates to labelling: it is unclear how the external hospital’s data were annotated, who performed the annotations, or whether definitions aligned with those used at the development site. Performance metrics are reported without confidence intervals, limiting the reader’s ability to assess statistical significance. The manuscript would also benefit from a more detailed analysis of *why* performance degraded at the external site, including example misclassifications and qualitative differences in triage language. Vocabulary and divergence analyses are presented but require deeper interpretation and clearer visualisation. Threshold selection could be better justified, particularly in the context of recalibrating for a new site. Finally, the authors should consider discussing subgroup or fairness-related performance, given differences in patient populations.

Minor areas for improvement include streamlining the Methods section, clarifying the rationale for the selected feature set, improving figure readability, and correcting minor spelling inconsistencies.

Despite these issues, this is a strong and insightful manuscript. The conceptual separation of the NLP normalisation pipeline from the classifier is particularly commendable, and the temporal analysis is a meaningful contribution. With clearer reporting and a deeper examination of dataset shift, the paper could become a valuable reference for researchers developing portable AI systems in healthcare.

See key guidance and suggestions below:

Title: Consider shortening for impact:

Abstract – Background

• “Natural language–based machine learning models may not be portable…”

→ Clarify what aspect: semantics, syntax, local jargon, EHR structure.

• Add one sentence describing why self-harm detection matters (clinical significance).

Abstract – Methods

• Explicitly state whether the external validation dataset was manually annotated.

• Include 95% confidence intervals for performance measures.

• Place the PR-AUC results into clearer comparative format:

o “Baseline RMH retrospective data: PR-AUC = 0.93 (CI…)”

o “Temporal external data…” etc.

• Strengthen causal phrasing:

o Instead of “suggests”, consider: “Performance degradation is primarily explained by linguistic domain shift and changes in triage documentation.”

INTRODUCTION

Paragraph 1

• Add 1–2 citations for global burden of self-harm and ED presentation rates.

• Sentence suggestion:

“Self-harm accounts for approximately ___% of ED mental health presentations… [ref]”

Paragraph 2 (AI Context)

• Replace vague: “Real-world performance may differ substantially.”

With:

“Real-world performance often degrades due to dataset shift in language, workflow, and patient demographics.”

Paragraph 3 (Gaps)

• Clarify the three axes of dataset shift you assess: Temporal/Geographic/Workflow/EHR system change

Final Paragraph (Study Aims)

• Rephrase for clarity and structure:

“We aimed to assess (1) temporal robustness of the model during prospective validation at RMH, and (2) geographic portability at a regional hospital (LRH). We also examined whether differences in local linguistic patterns, documentation practices, or preprocessing vocabulary contributed to performance decline.”

METHODS

• Add clearer demographic descriptions of RMH and LRH (population served, urban vs rural, proportion of Indigenous patients, etc.).

• Provide exact sample sizes with breakdowns:

o Total triage notes

o Total labelled cases

o Prevalence at each site

Major needed clarification.

Add a subsection titled “Annotation and Ground Truth Labelling”:

• Who annotated?

• Training and calibration?

• Inter-rater agreement (κ score)?

• Consistency between RMH and LRH labels?

Normalization Pipeline

• List key steps in a figure or flowchart rather than a long paragraph.

• Provide examples of:

o original triage text

o normalized text output

o common misspellings captured

Classifier Component

• Add rationale for choosing LightGBM vs alternatives.

• Clarify whether:

o Term frequency or subword n-grams were included.

o Stop words were removed.

o Case sensitivity was preserved or removed.

Evaluation Metrics

• Add a sentence:

“All estimates were accompanied by 95% confidence intervals calculated using 1000 bootstrap iterations.”

Comparison of Linguistic Differences

• Move technical formulas (JS divergence, TF-IDF cosine similarity) into Supplementary Materials.

• Provide interpretation guidance:

o “JS divergence values closer to 0 indicate similarity, while values >0.2 indicate substantial shift.”

RESULTS

Descriptive Statistics

• Add a transparent cohort table .

o Provide: median age, sex distribution, self-harm prevalence, missingness patterns

Temporal Results

• Clarify whether the drop in performance correlates with:

o COVID-19 lockdown period/EHR migration/triage staff turnover

• Include:

“Figure X: PR curves with shaded confidence intervals.”

Geographic Validation

• Add sub-analysis exploring:

o differences in top keywords

o misclassified cases categorized by reason (e.g., ambiguous language, euphemisms)

Error Analysis

• Provide five anonymised example triage notes highlighting:

o true positive, false positive, false negative, mis-spellings not captured, domain-specific language

DISCUSSION

Opening Paragraph

• Remove repetition from the introduction about dataset shift.

• Consider reorganising the section:

1. Summary of main findings

2. Implications for NLP in healthcare

3. Why performance declined in external setting

4. What should happen next (calibration, retraining, small labelled dataset, etc.)

Consider adding subsections:

• Impact of EHR Migration (workflow shift)

• Sociolinguistic Variability Across Regions

• Role of Fine-Tuning with Small Local Dataset

• Fairness Considerations (e.g., Indigenous patient language patterns)

Strengthen policy relevance:

“Health systems must consider ongoing monitoring, recalibration, and site-specific tuning before deploying NLP-based surveillance tools.”

CONCLUSION

Revise and consider:

“Future work will evaluate lightweight retraining, rapid calibration using small labelled batches, and deployment workflows to ensure equitable self-harm detection across diverse ED settings.”

FIGURES / TABLES

Figures

• Ensure all axes are clearly labelled with metric and CI.

o Improve readability using a consistent color palette, larger font, shading for confidence intervals

Tables

Consider adding a new Table 1 (Cohort Characteristics) and Table 2 (Model Performance) with 95% CI.

**********

what does this mean?). If published, this will include your full peer review and any attached files.

**Do you want your identity to be public for this peer review?** If you choose “no”, your identity will remain anonymous but your review may still be made public.

For information about this choice, including consent withdrawal, please see our Privacy Policy

Reviewer #1: No

Reviewer #2: **Yes:**  Godwin Ogbole

**********

**Figure resubmission:**

**Reproducibility:**

To enhance the reproducibility of your results, we recommend that authors of applicable studies deposit laboratory protocols in protocols.io, where a protocol can be assigned its own identifier (DOI) such that it can be cited independently in the future. Additionally, PLOS ONE offers an option to publish peer-reviewed clinical study protocols. Read more information on sharing protocols at https://plos.org/protocols?utm_medium=editorial-email&utm_source=authorletters&utm_campaign=protocols

# Author comment: Author response to Decision Letter 1

**DOI:** 10.1371/journal.pdig.0001667.r002

4 Mar 2026

Attachment Submitted filename: Rozova Self-harm portability Response to reviewers.pdf

# Peer review: Decision Letter 1

**DOI:** 10.1371/journal.pdig.0001667.r003

**Authors:** Laura Sbaffi, Phat Kim Huynh

26 May 2026

Response to Reviewers Revised Manuscript with Track Changes Manuscript **Journal Requirements:**

1. Please amend your detailed Financial Disclosure statement. This is published with the article. It must therefore be completed in full sentences and contain the exact wording you wish to be published.

i. Please clarify all sources of financial support for your study. List the grants, grant numbers, and organizations that funded your study, including funding received from your institution. Please note that suppliers of material support, including research materials, should be recognized in the Acknowledgements section rather than in the Financial Disclosure.

ii. State the initials, alongside each funding source, of each author to receive each grant. For example: "This work was supported by the National Institutes of Health (####### to AM; ###### to CJ) and the National Science Foundation (###### to AM)."

iii. State what role the funders took in the study. If the funders had no role in your study, please state: “The funders had no role in study design, data collection and analysis, decision to publish, or preparation of the manuscript.”

iv. If any authors received a salary from any of your funders, please state which authors and which funders.

2. We note that you have indicated that there are restrictions to data sharing for this study. For studies involving human research participant data or other sensitive data, we encourage authors to share de-identified or anonymized data. However, when data cannot be publicly shared for ethical reasons, we allow authors to make their data sets available upon request. For information on unacceptable data access restrictions, please see http://journals.plos.org/plosone/s/data-availability#loc-unacceptable-data-access-restrictions.

Before we proceed with your manuscript, please address the following prompts:

a) If there are ethical or legal restrictions on sharing a de-identified data set, please explain them in detail (e.g., data contain potentially identifying or sensitive patient information, data are owned by a third-party organization, etc.) and who has imposed them (e.g., a Research Ethics Committee or Institutional Review Board, etc.). Please also provide contact information for a data access committee, ethics committee, or other institutional body to which data requests may be sent.

b) If there are no restrictions, please upload the minimal anonymized data set necessary to replicate your study findings to a stable, public repository and provide us with the relevant URLs, DOIs, or accession numbers. Please see http://www.bmj.com/content/340/bmj.c181.long for guidelines on how to de-identify and prepare clinical data for publication. For a list of recommended repositories, please see https://journals.plos.org/plosone/s/recommended-repositories. You also have the option of uploading the data as Supporting Information files, but we would recommend depositing data directly to a data repository if possible.

Please update your Data Availability statement in the submission form accordingly.

3. Some material included in your submission may be copyrighted. According to PLOS’s copyright policy, authors who use figures or other material (e.g., graphics, clipart, maps) from another author or copyright holder must demonstrate or obtain permission to publish this material under the Creative Commons Attribution 4.0 International (CC BY 4.0) License used by PLOS journals. Please closely review the details of PLOS’s copyright requirements here: PLOS Licenses and Copyright. If you need to request permissions from a copyright holder, you may use PLOS's Copyright Content Permission form.

Please respond directly to this email or email the journal office and provide any known details concerning your material's license terms and permissions required for reuse, even if you have not yet obtained copyright permissions or are unsure of your material's copyright compatibility.

Potential Copyright Issues:

a. Figures 1 and 3: Please confirm whether you drew the images / clip-art within the figure panels by hand. If you did not draw the images, please provide (a) a link to the source of the images or icons and their license / terms of use; or (b) written permission from the copyright holder to publish the images or icons under our CC-BY 4.0 license. Alternatively, you may replace the images with open source alternatives. See these open source resources you may use to replace images / clip-art:

- https://commons.wikimedia.org

- https://openclipart.org/

4. Please upload separate figure files in .tif or .eps format. Also, remove the figures from your manuscript file but keep the legends.

For more information about figure files please see our guidelines:

https://journals.plos.org/digitalhealth/s/figures

https://journals.plos.org/digitalhealth/s/figures#loc-file-requirements

**Additional Editor Comments (if provided):** **Reviewers' Comments:**

**Comments to the Author**

Reviewer #3: All comments have been addressed

Reviewer #4: All comments have been addressed

Reviewer #5: All comments have been addressed

**********

publication criteria?

Reviewer #3: Yes

Reviewer #4: Yes

Reviewer #5: Yes

**********

3. Has the statistical analysis been performed appropriately and rigorously?

Reviewer #3: Yes

Reviewer #4: Yes

Reviewer #5: Yes

**********

4. Have the authors made all data underlying the findings in their manuscript fully available (please refer to the Data Availability Statement at the start of the manuscript PDF file)?

The PLOS Data policy

Reviewer #3: Yes

Reviewer #4: Yes

Reviewer #5: Yes

**********

5. Is the manuscript presented in an intelligible fashion and written in standard English?

Reviewer #3: Yes

Reviewer #4: Yes

Reviewer #5: Yes

**********

**Reviewer #3:**  This is an excellent, well-executed validation study that makes a timely and significant contribution to the field of clinical AI. The authors have performed a rigorous and comprehensive assessment of their previously developed NLP system for detecting self-harm in emergency department triage notes. The study robustly addresses the critical question of model portability across both time and different healthcare settings. The revisions made in response to the reviewers' comments are thorough and have substantially strengthened the manuscript's clarity, methodological rigour, and depth of analysis. The work is now suitable for publication in its current form.

- The following minus notes would be good to address：

The observed confusion between self-harm and suicidal ideation in model predictions is an interesting finding. Further brief reflection on how this might be managed in an operational public health surveillance or clinical decision-support context could be a valuable addition to future work.

- The logical next steps for clinical implementation (e.g., a feasibility pilot within the electronic health record system) are alluded to. A slightly more concrete outline of this translational pathway could further guide the research community.

**Reviewer #4:**  This manuscript presents a valuable study on self-harm surveillance. Building on prior work, the authors rigorously assessed the portability and temporal robustness of the self-harm classification model through prospective and external validation. The findings indicate that linguistic domain shift and differing presentations of self-harm contribute to a notable decline in positive predictive value (PPV). The study supports the development of a national suicide prevention system and offers valuable enlightening insights for peers carrying out other relevant similar researches.

The methods and technical approach are sound. The performance metrics are satisfactory under the evaluated conditions. Minor grammatical or stylistic issues do not detract from the manuscript.

I recommend discussing local linguistic patterns and differences in self-harm presentations, which may explain the drop in PPV. Potential dataset biases, including patient demographics, regional language usage, or living conventions, could be elaborated. Such analysis would clarify factors underlying reduced model performance, and is seems more important for readers from diverse national and cultural contexts.

**Reviewer #5:**  This is a revised manuscript that addresses a practical and clinically meaningful question: Whether AI self-harm detection model is generalizable across hospital sites. The authors have addressed most of the earlier concerns from previous reviewers and I can see that the revised version is substantially improved. Here, I have several comments that may further help improve the integrity and quality of the work.

1. Please clarify whether the clinical labels distinguish “current self-harm presentation” from “history of self-harm.” The false-positive example suggests that the model may classify a note as positive when self-harm is mentioned only as history. It would be helpful to state clearly in the annotation section that the positive label refers to self-harm related to the current ED presentation, not past history alone.

2. The part of error analysis is very useful, as it points out a few cases that we still require manual review. I’d mention this and discuss a bit more in the limitation paragraph in Discussion section.

3. The added confidence bands and feature-importance plots are useful, but these figures may become visually dense. Please make sure that the axis labels, legends, and font sizes remain readable and consistent in the final journal format (e.g. make Figure 6BCDEF font sizes the same as in Panel A).

4. Also some y-limit axes could be zoomed in, otherwise it is very hard to eyeball the differences across different sets. For example, Figure 4 G and H.

5. I found the thresholding strategy a little hard to follow. The manuscript says the final probability threshold was selected by maximizing F1 score, and later gives an example where a case with predicted probability 0.3215 was classified as negative because it fell just below the threshold of 0.3225. However, it is not clear to me whether this threshold was chosen only once using the RMH development data and then applied unchanged to the RMH prospective and LRH external validation datasets, or whether it was adjusted for LRH. I suggest the authors state this explicitly in the Methods.

**********

what does this mean?). If published, this will include your full peer review and any attached files.

**Do you want your identity to be public for this peer review?** If you choose “no”, your identity will remain anonymous but your review may still be made public.

For information about this choice, including consent withdrawal, please see our Privacy Policy

Reviewer #3: No

Reviewer #4: No

Reviewer #5: No

**********

**Figure resubmission:**

**Reproducibility:**

To enhance the reproducibility of your results, we recommend that authors of applicable studies deposit laboratory protocols in protocols.io, where a protocol can be assigned its own identifier (DOI) such that it can be cited independently in the future. Additionally, PLOS ONE offers an option to publish peer-reviewed clinical study protocols. Read more information on sharing protocols at https://plos.org/protocols?utm_medium=editorial-email&utm_source=authorletters&utm_campaign=protocols

# Author comment: Author response to Decision Letter 2

**DOI:** 10.1371/journal.pdig.0001667.r004

17 Jun 2026

Attachment Submitted filename: Rozova Self-harm portability Response to reviewers round 2.pdf

# Peer review: Decision Letter 2

**DOI:** 10.1371/journal.pdig.0001667.r005

**Authors:** Laura Sbaffi, Phat Kim Huynh

27 Jul 2026

Temporal and cross-site validation of an AI system for self-harm detection

PDIG-D-25-00473R2

Dear Miss Rozova,

We are pleased to inform you that your manuscript 'Temporal and cross-site validation of an AI system for self-harm detection' has been provisionally accepted for publication in PLOS Digital Health.

Before your manuscript can be formally accepted you will need to complete some formatting changes, which you will receive in a follow-up email from a member of our team.

Please note that your manuscript will not be scheduled for publication until you have made the required changes, so a swift response is appreciated.

IMPORTANT: The editorial review process is now complete. PLOS will only permit corrections to spelling, formatting or significant scientific errors from this point onwards. Requests for major changes, or any which affect the scientific understanding of your work, will cause delays to the publication date of your manuscript.

If your institution or institutions have a press office, please notify them about your upcoming paper to help maximize its impact. If they'll be preparing press materials, please inform our press team as soon as possible -- no later than 48 hours after receiving the formal acceptance. Your manuscript will remain under strict press embargo until 2 pm Eastern Time on the date of publication. For more information, please contact digitalhealth@plos.org.

Thank you again for supporting Open Access publishing; we are looking forward to publishing your work in PLOS Digital Health.

Best regards,

Phat Kim Huynh, Ph.D.

Guest Editor

PLOS Digital Health

***********************************************************

**Additional Editor Comments (if provided):**

**Reviewer Comments (if any, and for reference):**

Reviewer's Responses to Questions

**Comments to the Author**

Reviewer #3: (No Response)

Reviewer #4: All comments have been addressed

Reviewer #5: All comments have been addressed

**********

publication criteria?

Reviewer #3: (No Response)

Reviewer #4: Yes

Reviewer #5: Yes

**********

3. Has the statistical analysis been performed appropriately and rigorously?

Reviewer #3: (No Response)

Reviewer #4: Yes

Reviewer #5: Yes

**********

4. Have the authors made all data underlying the findings in their manuscript fully available (please refer to the Data Availability Statement at the start of the manuscript PDF file)?

The PLOS Data policy

Reviewer #3: (No Response)

Reviewer #4: No

Reviewer #5: Yes

**********

5. Is the manuscript presented in an intelligible fashion and written in standard English?

Reviewer #3: (No Response)

Reviewer #4: Yes

Reviewer #5: Yes

**********

Reviewer #3: The revisions made in response to the reviewers' comments are thorough and have substantially strengthened the manuscript's clarity, methodological rigour, and depth of analysis. The work is now suitable for publication in its current form.

Reviewer #4: The manuscript has addressed all comments.

Reviewer #5: I was a reviewer on the previous submission of this manuscript. The authors have addressed my concerns through this revision, which has greatly improved the rigor and clarity of the work. I appreciate their efforts and have no further major concerns.

**********

what does this mean?). If published, this will include your full peer review and any attached files.

**Do you want your identity to be public for this peer review?** If you choose “no”, your identity will remain anonymous but your review may still be made public.

For information about this choice, including consent withdrawal, please see our Privacy Policy

Reviewer #3: No

Reviewer #4: No

Reviewer #5: No

**********
